# Supplementary material for: Deciphering the Molecular Mechanisms of Reactive Metabolite Formation in the Mechanism-Based Inactivation of Cytochrome p450 1B1 by 8-Methoxypsoralen and Assessing the Driving Effect of phe268
Source: Molecules. 2024 Mar 22;29(7):1433. doi: 10.3390/molecules29071433 (PMC11012842; doi:10.3390/molecules29071433)
Supplement: Supplementary file 1 [file molecules-29-01433-s001.zip › molecules-2920926-supplementary.pdf]

Deciphering the Molecular Mechanisms of Reactive Metabolite Formation in the Mechanism-Based Inactivation of Cytochrome p450 1B1 by 8-Methoxypsoralen and Assessing the Driving Effect of phe268

Emadeldin M. Kamel [1], Maha A. Alwaili [2,*], Hassan A. Rudayni [3], Ahmed A. Allam [3,4] and Al Mokhtar Lamsabhi [5,6,*]

[1] Chemistry Department, Faculty of Science, Beni-Suef University, Beni-Suef 62514, Egypt; emad.abdelhameed@science.bsu.edu.eg
[2] Department of Biology, College of Science, Princess Nourah bint Abdulrahman University, P.O. Box 84428, Riyadh 11671, Saudi Arabia
[3] Department of Biology, College of Science, Imam Mohammad Ibn Saud Islamic University, Riyadh 11623, Saudi Arabia; harudayni@imamu.edu.sa (H.A.R.); ahmed.aliahmed@science.bsu.edu.eg (A.A.A.)
[4] Department of Zoology, Faculty of Science, Beni-Suef University, Beni-Suef 65211, Egypt
[5] Departamento de Química, Módulo 13, Universidad Autónoma de Madrid, Campus de Excelencia UAM-CSIC Cantoblanco, 28049 Madrid, Spain
[6] Institute for Advanced Research in Chemical Sciences (IAdChem), Universidad Autónoma de Madrid, 28049 Madrid, Spain
* Correspondence: maalwaele@pnu.edu.sa (M.A.A.); mokhtar.lamsabhi@uam.es (A.M.L.)

Table of Contents

Table S1. Relative energies (RE) (in kcal/mol) and atomic energies (in a.u.) at the B3LYP level of theory for the MBI of P450 by 8-MP through 8-OMe C-H hydroxylation (Path A)……….…………….……..………….S2

Table S2. Relative energies (RE) (in kcal/mol) and atomic energies (in a.u.) at the B3LYP level of theory for the MBI of P450 by 8-MP through C3=C4 epoxidation (Path B)…………………. …..………….…………..…..S2

Table S3. Relative energies (RE) (in kcal/mol) and atomic energies (in a.u.) at the B3LYP level of theory for the MBI of P450 by 8-MP through C4′=C5′ epoxidation (Path C)……………………… …………….……..….S3

Table S4. Relative energies (RE) (in kcal/mol) and atomic energies (in a.u.) at the B3LYP level of theory for the MBI of P450 by 8-MP through C4′=C5′ epoxidation directed by phenylalanine……………..……………..…..S3

Table S5. Relative energies (RE) (in kcal/mol) and atomic energies (in a.u.) at the B3LYP level of theory for various mechanisms of reactive metabolites reactions of 8-MP epoxide……………………..…..………….. S4

Cartesian coordinates of all structures represented in this study……………………………………………S5-66

Table S1. Relative energies (RE) (in kcal/mol) and atomic energies (in a.u.) at the B3LYP level of theory for the MBI of P450 by 8-MP through 8-OMe C-H hydroxylation (Path A)

	UB3LYP/BS1		UB3LYP/BS1+ZPE		UB3LYP/BS2//BS1		UB3LYP/BS2//BS1+ZPE		UB3LYP/BS2//BS1 +Bulk Polarity		UB3LYP/BS2//BS1+ZPE +Bulk Polarity	
	AE	RE	AE	RE	AE	RE	AE	RE	AE	RE	AE	RE
$^{2}RC_{A}$	-2348.25803400	0.00	-2347.79099000	0.00	-2349.27036700	0.00	-2348.80332300	0.00	-2349.28982200	0.00	-2348.82277800	0.00
$^{4}RC_{A}$	-2348.25809600	-0.04	-2347.79098300	0.00	-2349.27024300	0.08	-2348.80313000	0.12	-2349.28976100	0.04	-2348.82264800	0.08
$^{2}TS\text{-}H_{A}$	-2348.22260300	22.23	-2347.76256800	17.84	-2349.24116800	18.32	-2348.78113300	13.92	-2349.26273800	17.00	-2348.80270300	12.60
$^{4}TS\text{-}H_{A}$	-2348.22309100	21.93	-2347.76222000	18.05	-2349.24186500	17.89	-2348.78099400	14.01	-2349.26148500	17.78	-2348.80061400	13.91
$^{2}INT_{A}$	-2348.23259100	15.97	-2347.76564500	15.90	-2349.26212350	5.17	-2348.79517750	5.11	-2349.282412	4.65	-2348.81546600	4.59
$^{4}INT_{A}$	-2348.23173600	16.50	-2347.76801900	14.41	-2349.25818700	7.64	-2348.79447000	5.56	-2349.278685	6.99	-2348.81496800	4.90
$^{4}TS\text{-}reb_{A}$	-2348.22739600	19.23	-2347.76506000	16.27	-2349.25584300	9.11	-2348.79350700	6.16	-2349.275218	9.16	-2348.81288200	6.21
$^{2}P\text{-}OH_{A}$	-2348.33430900	-47.86	-2347.86364800	-45.59	-2349.36567400	-59.81	-2348.89501300	-57.54	-2349.388021	-61.62	-2348.91736000	-59.35
$^{4}P\text{-}OH_{A}$	-2348.32778000	-43.77	-2347.85897900	-42.66	-2349.34287300	-45.50	-2348.87407200	-44.40	-2349.367856	-48.97	-2348.89905500	-47.86

Table S2. Relative energies (RE) (in kcal/mol) and atomic energies (in a.u.) at the B3LYP level of theory for the MBI of P450 by 8-MP through C3=C4 epoxidation (Path B).

	UB3LYP/BS1		UB3LYP/BS1+ZPE		UB3LYP/BS2//BS1		UB3LYP/BS2//BS1+ZPE		UB3LYP/BS2//BS1 +Bulk Polarity		UB3LYP/BS2//BS1+ZPE +Bulk Polarity	
	AE	RE	AE	RE	AE	RE	AE	RE	AE	RE	AE	RE
$^{2}RC_{B}$	-2348.25616600	0.00	-2347.78953100	0.00	-2349.26933500	0.00	-2348.80270000	0.00	-2349.28974400	0.00	-2348.82310900	0.00
$^{4}RC_{B}$	-2348.25624200	-0.05	-2347.78953200	0.00	-2349.26924600	0.06	-2348.80253600	0.10	-2349.28967000	0.05	-2348.82296000	0.09
$^{2}TS\text{-}O_{B}$	-2348.22892500	17.09	-2347.76346100	16.36	-2349.24361400	16.14	-2348.77815000	15.41	-2349.26576000	15.05	-2348.80029600	14.32
$^{4}TS\text{-}O_{B}$	-2348.22716000	18.20	-2347.76134800	17.69	-2349.24311200	16.46	-2348.77730000	15.94	-2349.26337000	16.55	-2348.79755800	16.03
$^{2}INT_{B}$	-2348.27558100	-12.18	-2347.80661500	-10.72	-2349.31227500	-26.95	-2348.84330900	-25.48	-2349.331456	-26.17	-2348.86249000	-24.71
$^{4}INT_{B}$	-2348.27412200	-11.27	-2347.80680200	-10.84	-2349.29047810	-13.27	-2348.82315810	-12.84	-2349.309145	-12.17	-2348.84182500	-11.74
$^{2}P\text{-}epo_{B}$	-2348.27719900	-13.20	-2347.80839500	-11.84	-2349.31641200	-29.54	-2348.84760800	-28.18	-2349.335972	-29.01	-2348.86716810	-27.65
$^{4}P\text{-}epo_{B}$	-2348.25089400	3.31	-2347.78447500	3.17	-2349.29110100	-13.66	-2348.82468200	-13.79	-2349.312442	-14.24	-2348.84602300	-14.38

Table S3. Relative energies (RE) (in kcal/mol) and atomic energies (in a.u.) at the B3LYP level of theory for the MBI of P450 by 8-MP through C4′=C5′ epoxidation (Path C).

	UB3LYP/BS1		UB3LYP/BS1+ZPE		UB3LYP/BS2//BS1		UB3LYP/BS2//BS1+ZPE		UB3LYP/BS2//BS1 +Bulk Polarity		UB3LYP/BS2//BS1+ZPE+ Bulk Polarity	
	AE	RE	AE	RE	AE	RE	AE	RE	AE	RE	AE	RE
^{2}RC$_{C}$	-2348.25429000	0.00	-2347.78769300	0.00	-2349.26984200	0.00	-2348.80324500	0.00	-2349.29278500	0.00	-2348.82618800	0.00
^{4}RC$_{C}$	-2348.25420700	0.05	-2347.78767700	0.01	-2349.26963800	0.13	-2348.80310800	0.09	-2349.29262200	0.10	-2348.82609200	0.06
^{2}TS-O$_{C}$	-2348.23212800	13.91	-2347.76664600	13.21	-2349.24836700	13.48	-2348.78288500	12.78	-2349.27086100	13.76	-2348.80537900	13.06
^{4}TS-O$_{C}$	-2348.23269600	13.55	-2347.76677100	13.13	-2349.24994600	12.48	-2348.78402100	12.06	-2349.27096400	13.69	-2348.80503900	13.27
^{2}INT$_{C}$	-2348.28283400	-17.91	-2347.81413900	-16.60	-2349.31473900	-28.17	-2348.84604400	-26.86	-2349.337247	-27.90	-2348.86855200	-26.58
^{4}INT$_{C}$	-2348.26348700	-5.77	-2347.79768900	-6.27	-2349.28867300	-11.82	-2348.82287500	-12.32	-2349.310033	-10.82	-2348.84423500	-11.32
^{2}P-epo$_{C}$	-2348.28322500	-18.16	-2347.81442100	-16.77	-2349.31972200	-31.30	-2348.85091800	-29.92	-2349.340873	-30.18	-2348.87206900	-28.79
^{4}P-epo$_{C}$	-2348.26000500	-3.59	-2347.79348800	-3.64	-2349.29898100	-18.29	-2348.83246400	-18.34	-2349.321029	-17.72	-2348.85451200	-17.77

Table S4. Relative energies (RE) (in kcal/mol) and atomic energies (in a.u.) at the B3LYP level of theory for the MBI of P450 by 8-MP through C4′=C5′ epoxidation directed by phenylalanine.

	UB3LYP/BS1		UB3LYP/BS1+ZPE		UB3LYP/BS2//BS1		UB3LYP/BS2//BS1+ZPE		UB3LYP/BS2//BS1 +Bulk Polarity		UB3LYP/BS2//BS1+ZPE+ Bulk Polarity	
	AE	RE	AE	RE	AE	RE	AE	RE	AE	RE	AE	RE
^{2}RC$_{ph}$	-2902.91079900	0.00	-2902.25231500	0.00	-2904.24097400	0.00	-2903.58249000	0.00	-2904.26585800	0.00	-2903.60737400	0.00
^{4}RC$_{ph}$	-2902.91046100	0.21	-2902.25197300	0.21	-2904.24059400	0.24	-2903.58210600	0.24	-2904.26552000	0.21	-2903.60703200	0.21
^{2}TS-O$_{ph}$	-2902.89136000	12.20	-2902.23419000	11.37	-2904.21910400	13.72	-2903.56193400	12.90	-2904.24520000	12.96	-2903.58803000	12.14
^{4}TS-O$_{ph}$	-2902.88865300	13.90	-2902.23215300	12.65	-2904.21844000	14.14	-2903.56194000	12.90	-2904.24485600	13.18	-2903.58835600	11.93
^{2}INT$_{ph}$	-2902.92467900	-8.71	-2902.26374500	-7.17	-2904.27935500	-24.08	-2903.61842100	-22.55	-2904.304481	-24.24	-2903.64354700	-22.70
^{4}INT$_{ph}$	-2902.92920600	-11.55	-2902.26981900	-10.98	-2904.25491300	-8.75	-2903.59552600	-8.18	-2904.284211	-11.52	-2903.62482400	-10.95
^{2}P$_{ph}$	-2902.93007600	-12.10	-2902.26959800	-10.85	-2904.28200300	-25.75	-2903.62152500	-24.49	-2904.306954	-25.79	-2903.64647600	-24.54
^{4}P$_{ph}$	-2902.91841400	-4.78	-2902.25957200	-4.55	-2904.28258200	-26.11	-2903.62374000	-25.88	-2904.311705	-28.77	-2903.65286300	-28.54

Table S5. Relative energies (RE) (in kcal/mol) and atomic energies (in a.u.) at the B3LYP level of theory for various mechanisms of reactive metabolites reactions of 8-MP epoxide

OMe addition	EE+ZPE (6-311G**)	RE (kcal/mol)	SP EE (6-311+G**) (SCRF=chlorobenzene)	SP EE(SCRF)+ZPE	RE (kcal/mol)
epoxide+HOMe	-953.878719	0.00	-954.152311	-953.922	0.00
TS_{OMe}	-953.832932	28.73	-954.104811	-953.875	29.53
P_{OMe}	-953.913907	-22.08	-954.18727	-953.952	-19.39
SMe addition					
epoxide+HSMe	-1276.869132	0.00	-1277.128964	-1276.9	0.00
TS_{SMe}	-1276.810965	36.50	-1277.073203	-1276.85	33.68
P_{SMe}	-1276.905445	-22.79	-1277.16977	-1276.94	-22.07
Phenylalanine addition					
epoxide+phe	-1392.929024	0.00	-1393.350828	-1392.98	0.00
TS_{Phe}	-1392.891489	23.55	-1393.316239	-1392.94	25.71
P_{Phe}	-1392.965362	-22.80	-1393.381634	-1393.01	-16.90
Nonenzymatic Ring opening					
epoxide	-838.172441	0.00	-838.382959	-838.203	0.00
TS_{ro}	-838.146167	16.49	-838.358035	-838.181	13.79
P_{ro}	-838.159207	8.30	-838.368675	-838.191	7.69
Diol formation pathway					
epoxide+HOH	-914.598564	0.00	-914.84745	-914.647	0.00
TS_{diol}	-914.54992	30.52	-914.798918	-914.597	31.00
P_{diol}	-914.641697	-27.07	-914.886673	-914.679	-20.39
TS_{r1}	-914.527504	44.59	-914.76621	-914.564	51.71
P_{r2}	-914.634892	-22.80	-914.873837	-914.668	-13.61
TS_{r2}	-914.574756	14.94	-914.812524	-914.612	21.90
P_{r2}	-914.641915	-27.20	-914.885567	-914.68	-21.25

Cartesian coordinates of all structures represented in this study.

Path A

^2RC_A

Fe	-2.18908	-0.13058	0.03266
O	-1.12155	0.79172	0.89202
S	-3.70858	-1.42718	-1.61732
H	-2.88099	-2.51305	-1.83294
N	-1.39793	-1.87803	0.6561
C	0.01648	-3.69713	0.91836
C	-0.86818	-3.72374	1.95913
H	0.81917	-4.38579	0.69926
H	-0.93367	-4.43869	2.76631
N	-3.55581	-0.22267	1.50691
C	-3.61283	-1.15376	2.52943
C	-4.6843	-0.822	3.44262
C	-5.27501	0.31441	2.9671
H	-4.93997	-1.39346	4.32292
H	-6.11163	0.85929	3.37941
N	-3.15893	1.45432	-0.76022
C	-4.23404	2.13458	-0.20273
C	-2.82031	2.15219	-1.90676
C	-4.57512	3.27539	-1.02436
C	-3.70207	3.28764	-2.07383
H	-5.38066	3.96351	-0.81354
H	-3.65062	3.98728	-2.89521
N	-1.01857	-0.20994	-1.61356
C	0.12096	0.38605	-3.55117
H	0.3755	0.95306	-4.43452
C	-0.9599	0.72154	-2.64814
C	-0.31584	-2.54491	0.10952
C	-1.74542	-2.58506	1.79721
C	-4.56888	0.68287	1.75968
C	-1.79167	1.81854	-2.77797
H	-1.63509	2.46504	-3.63494
C	0.35502	-2.17968	-1.05262
H	1.20416	-2.77859	-1.36405
C	-2.77541	-2.2522	2.66269
H	-2.93736	-2.90075	3.51731
C	-4.88461	1.77863	0.9656
H	-5.71221	2.40296	1.28594
C	0.73092	-0.73015	-3.05926
H	1.5945	-1.25953	-3.43142
C	0.02653	-1.09986	-1.85102
C	5.55419	1.56444	1.69137
C	6.11352	0.31081	1.44705
C	5.49968	-0.51661	0.48814

C	4.34035	-0.07313	-0.2024
C	3.75982	1.19223	0.00688
C	4.4049	1.97399	0.96876
H	6.88831	-2.1807	0.67429
H	6.9978	-0.03105	1.97303
C	5.99381	-1.83378	0.16537
C	4.16447	-2.20687	-1.4208
C	5.37192	-2.63338	-0.73996
H	5.73214	-3.62391	-0.9843
O	3.7168	-0.90013	-1.12936
O	3.48861	-2.87384	-2.21482
O	2.70236	1.56682	-0.77574
C	1.59267	2.35573	-0.19238
H	0.97787	2.61954	-1.05037
H	1.97246	3.24969	0.30339
O	4.04386	3.26432	1.35813
C	4.979	3.65322	2.33999
H	4.82505	4.63807	2.74466
C	5.89025	2.67518	2.56509
H	6.71164	2.71679	3.26302
H	1.00969	1.73237	0.48962

^{4}RC$_{A}$

Fe	-2.18304	-0.1332	0.02461
O	-1.13072	0.78576	0.91087
S	-3.71159	-1.43731	-1.58033
H	-2.88165	-2.51864	-1.80883
N	-1.3909	-1.87518	0.66539
C	0.02399	-3.69129	0.94569
C	-0.86583	-3.71258	1.98209
H	0.82847	-4.38038	0.73469
H	-0.93439	-4.42253	2.79342
N	-3.55067	-0.21144	1.50523
C	-3.60972	-1.1368	2.53216
C	-4.67669	-0.79418	3.44716
C	-5.26192	0.34339	2.96783
H	-4.93266	-1.35933	4.33143
H	-6.09375	0.89558	3.3801
N	-3.14922	1.44886	-0.77432
C	-4.22293	2.13488	-0.22001
C	-2.81657	2.13347	-1.93043
C	-4.56721	3.26649	-1.05292
C	-3.69902	3.26682	-2.10652
H	-5.37203	3.95677	-0.84646
H	-3.65142	3.95699	-2.93609
N	-1.01104	-0.22279	-1.61291
C	0.11657	0.34644	-3.5657

H	0.36579	0.90143	-4.45815
C	-0.95941	0.69371	-2.66156
C	-0.30639	-2.54477	0.12787
C	-1.7436	-2.57567	1.80922
C	-4.5575	0.70083	1.75577
C	-1.79133	1.78926	-2.8016
H	-1.63831	2.42487	-3.66732
C	0.36441	-2.18784	-1.03686
H	1.21406	-2.78815	-1.34412
C	-2.77607	-2.23774	2.66973
H	-2.94083	-2.88239	3.5268
C	-4.87097	1.79133	0.95344
H	-5.69592	2.42103	1.27012
C	0.72989	-0.76252	-3.06188
H	1.59173	-1.29654	-3.43148
C	0.03212	-1.11671	-1.84532
C	5.5421	1.58496	1.68231
C	6.10826	0.33328	1.44391
C	5.49743	-0.50304	0.49081
C	4.33419	-0.07023	-0.19986
C	3.74646	1.19275	0.00379
C	4.38894	1.98377	0.95984
H	6.89625	-2.15786	0.68334
H	6.9956	-0.00042	1.96997
C	5.99887	-1.81891	0.1741
C	4.16942	-2.21112	-1.40732
C	5.38041	-2.62687	-0.72614
H	5.74624	-3.61643	-0.96601
O	3.71395	-0.90578	-1.12132
O	3.4966	-2.886	-2.19719
O	2.68479	1.55594	-0.77855
C	1.5745	2.3469	-0.19965
H	0.95631	2.60056	-1.05825
H	1.95279	3.24667	0.28657
O	4.02134	3.27422	1.34268
C	4.95632	3.67409	2.32028
H	4.79757	4.66036	2.71962
C	5.87362	2.70258	2.54898
H	6.69626	2.75285	3.24486
H	0.99574	1.72836	0.49044

^{2}TS-H$_{A}$

Fe	1.92662	-0.25835	-0.11892
O	0.83939	1.15127	0.02999
S	3.41603	-2.1279	-0.77325
H	2.67834	-3.16599	-0.23445
N	0.60606	-1.30169	-1.23265

C	-1.15738	-2.66274	-1.89514
C	-0.68641	-2.0393	-3.0152
H	-1.96879	-3.36892	-1.80082
H	-1.03472	-2.13817	-4.03318
N	2.6524	0.62056	-1.79784
C	2.15543	0.49299	-3.07904
C	2.89836	1.34576	-3.98822
C	3.84967	1.98413	-3.24736
H	2.70833	1.42599	-5.04866
H	4.59459	2.69298	-3.57866
N	3.38697	0.60864	0.96023
C	4.33835	1.50139	0.50491
C	3.60639	0.47484	2.31914
C	5.17723	1.93376	1.60209
C	4.7255	1.2976	2.7243
H	5.99888	2.62918	1.51062
H	5.10316	1.36916	3.73392
N	1.26469	-1.21814	1.52262
C	1.00136	-1.92578	3.72155
H	1.1926	-2.01333	4.78129
C	1.77082	-1.10356	2.81148
C	-0.36077	-2.19256	-0.78235
C	0.40366	-1.18017	-2.60355
C	3.69367	1.52932	-1.87892
C	2.85773	-0.32224	3.17633
H	3.14743	-0.33325	4.22236
C	-0.55782	-2.55717	0.54026
H	-1.38489	-3.22614	0.75101
C	1.10953	-0.34189	-3.4507
H	0.825	-0.34454	-4.49805
C	4.47891	1.93135	-0.81074
H	5.27317	2.64198	-1.01602
C	0.0336	-2.54361	2.98095
H	-0.72934	-3.23291	3.31113
C	0.20733	-2.11116	1.61145
C	-4.58919	2.7192	-0.10057
C	-5.41915	1.74495	-0.65407
C	-5.09767	0.39155	-0.44015
C	-3.95293	0.04142	0.32245
C	-3.10337	1.00243	0.90032
C	-3.45834	2.33133	0.6619
H	-6.77486	-0.44233	-1.54758
H	-6.29382	2.00897	-1.23765
C	-5.88721	-0.69309	-0.97454
C	-4.35327	-2.36243	-0.04229
C	-5.54422	-1.99324	-0.7843

H	-6.12515	-2.8116	-1.18858
O	-3.62088	-1.28843	0.52343
O	-3.91271	-3.50212	0.13568
O	-2.0623	0.54761	1.67271
C	-0.87852	1.29849	1.89853
H	-0.36061	0.86087	2.74505
H	-1.00534	2.37815	1.89001
O	-2.79768	3.46566	1.12766
C	-3.52498	4.57302	0.63926
H	-3.12374	5.53206	0.9154
C	-4.59351	4.17164	-0.09119
H	-5.31221	4.81342	-0.5758
H	0.04916	1.11307	0.82079

^{4}TS-H$_{A}$

Fe	1.94896	-0.27503	-0.17358
O	0.78626	1.03688	0.00445
S	3.45094	-2.13729	-0.16406
H	2.62342	-3.05513	-0.78198
N	0.96794	-1.05052	-1.73921
C	-0.58659	-2.17043	-3.04361
C	0.22866	-1.47647	-3.8929
H	-1.42132	-2.81113	-3.28832
H	0.19088	-1.43124	-4.97164
N	3.07179	0.81917	-1.39437
C	3.0089	0.85584	-2.7793
C	3.97822	1.80263	-3.28995
C	4.62654	2.33376	-2.21321
H	4.12936	2.01837	-4.33767
H	5.4182	3.06884	-2.20235
N	3.04362	0.3375	1.41189
C	4.05174	1.28419	1.39299
C	2.89645	-0.05	2.73706
C	4.55729	1.48883	2.73243
C	3.84874	0.66309	3.55953
H	5.35184	2.1742	2.98951
H	3.94693	0.54019	4.62837
N	0.83637	-1.44468	1.08709
C	-0.0259	-2.48071	2.97472
H	-0.12949	-2.75258	4.01513
C	0.99226	-1.58743	2.45794
C	-0.1292	-1.90386	-1.69781
C	1.18823	-0.76708	-3.07625
C	4.07086	1.71057	-1.02988
C	1.95346	-0.94535	3.22274
H	1.96233	-1.14801	4.28852
C	-0.7035	-2.43829	-0.55443

H	-1.56673	-3.08333	-0.67301
C	2.14864	0.10768	-3.56412
H	2.21026	0.23546	-4.63951
C	4.51996	1.93582	0.26003
H	5.31869	2.65766	0.3931
C	-0.7953	-2.87124	1.91719
H	-1.66943	-3.50466	1.90395
C	-0.2447	-2.234	0.7385
C	-4.55296	2.73907	-0.25064
C	-5.39217	1.75327	-0.76883
C	-5.10042	0.40662	-0.48175
C	-3.97583	0.07453	0.31785
C	-3.1185	1.04848	0.86281
C	-3.44326	2.36987	0.55105
H	-6.77357	-0.4519	-1.57679
H	-6.25135	2.00344	-1.38084
C	-5.90085	-0.68929	-0.97578
C	-4.41553	-2.33763	0.06755
C	-5.58599	-1.98421	-0.71425
H	-6.1759	-2.81115	-1.08687
O	-3.66958	-1.24885	0.58741
O	-4.00181	-3.47435	0.31287
O	-2.10087	0.61296	1.67472
C	-0.90422	1.34848	1.88952
H	-0.40939	0.93055	2.76078
H	-1.01462	2.42986	1.84836
O	-2.76748	3.51369	0.96913
C	-3.46408	4.60806	0.41104
H	-3.04827	5.5723	0.64418
C	-4.5281	4.18982	-0.31653
H	-5.22523	4.81921	-0.84678
H	0.01799	1.08587	0.84323

^{2}INT$_{A}$

Fe	-2.18995	0.0868	0.01941
O	-1.14207	-0.82774	-0.8719
S	-3.77898	1.79449	1.14908
H	-2.84766	2.42287	1.95444
N	-1.38644	1.84387	-0.58409
C	0.05408	3.65049	-0.82482
C	-0.81277	3.68484	-1.87902
H	0.85958	4.33224	-0.59464
H	-0.85806	4.40159	-2.68594
N	-3.55506	0.22404	-1.4629
C	-3.57551	1.14735	-2.49496
C	-4.64513	0.83418	-3.41782
C	-5.27177	-0.28135	-2.94148

H	-4.87592	1.40611	-4.30462
H	-6.11787	-0.80629	-3.36032
N	-3.18937	-1.48151	0.78803
C	-4.27409	-2.13256	0.22827
C	-2.88052	-2.1787	1.94204
C	-4.65139	-3.263	1.0479
C	-3.79011	-3.29112	2.10809
H	-5.47098	-3.9323	0.83085
H	-3.76363	-3.98816	2.93288
N	-1.0022	0.12425	1.64591
C	0.11275	-0.49784	3.58715
H	0.34973	-1.07082	4.47161
C	-0.9753	-0.80092	2.68007
C	-0.29185	2.49763	-0.02356
C	-1.70387	2.55455	-1.73789
C	-4.58887	-0.66379	-1.72489
C	-1.85277	-1.86309	2.82173
H	-1.72483	-2.50352	3.68821
C	0.38131	2.09614	1.11716
H	1.23666	2.67909	1.44164
C	-2.70942	2.22315	-2.62826
H	-2.84021	2.86339	-3.49419
C	-4.92236	-1.75516	-0.94009
H	-5.76134	-2.36306	-1.26241
C	0.75008	0.60422	3.09582
H	1.62257	1.11717	3.47022
C	0.04864	0.99076	1.89022
C	5.56508	-1.51928	-1.72883
C	6.12796	-0.2739	-1.45241
C	5.51613	0.53033	-0.47267
C	4.35546	0.07218	0.20574
C	3.77179	-1.18611	-0.03517
C	4.41469	-1.94453	-1.01694
H	6.90944	2.19486	-0.61468
H	7.01328	0.07879	-1.96938
C	6.0137	1.83729	-0.11539
C	4.18429	2.17441	1.47861
C	5.3937	2.61483	0.8103
H	5.75671	3.59758	1.08056
O	3.7332	0.87698	1.15277
O	3.5084	2.82201	2.28844
O	2.71425	-1.57652	0.73922
C	1.60487	-2.35574	0.14289
H	0.98968	-2.63294	0.99631
H	1.0218	-1.72185	-0.52915
H	1.98466	-3.24157	-0.36728

O	4.0504	-3.22343	-1.43958
C	4.98451	-3.5891	-2.4313
H	4.82799	-4.5627	-2.86142
C	5.89827	-2.60791	-2.631
H	6.71952	-2.63348	-3.32986

^{4}INT$_A$

Fe	-2.17612	0.22884	0.02743
O	-1.03346	-0.92868	-0.76203
S	-3.69241	1.66945	1.11139
H	-3.18365	2.86951	0.65374
N	-1.30965	1.82204	-0.85432
C	0.14307	3.55367	-1.35359
C	-0.72011	3.43391	-2.40665
H	0.95558	4.25423	-1.22691
H	-0.75305	4.01522	-3.3167
N	-3.41239	0.02471	-1.54
C	-3.42368	0.78922	-2.69481
C	-4.45043	0.30117	-3.59022
C	-5.06122	-0.74943	-2.96762
H	-4.66474	0.72258	-4.56158
H	-5.87719	-1.35973	-3.32638
N	-3.15566	-1.23128	1.00633
C	-4.21151	-1.98445	0.51799
C	-2.8731	-1.72287	2.27215
C	-4.61006	-2.95836	1.50912
C	-3.78872	-2.79582	2.58904
H	-5.41398	-3.6681	1.37871
H	-3.78619	-3.34617	3.51865
N	-0.90611	0.41745	1.59406
C	0.12395	0.16085	3.6533
H	0.32307	-0.24157	4.63593
C	-0.93764	-0.29821	2.78297
C	-0.21551	2.53981	-0.38833
C	-1.61699	2.34208	-2.1025
C	-4.41895	-0.91547	-1.6816
C	-1.84875	-1.29212	3.10153
H	-1.7555	-1.76736	4.07186
C	0.45756	2.3174	0.80299
H	1.30049	2.95522	1.04294
C	-2.60046	1.87064	-2.95799
H	-2.72572	2.3795	-3.90737
C	-4.79462	-1.8498	-0.73147
H	-5.61255	-2.51751	-0.97827
C	0.78305	1.16171	2.99853
H	1.64183	1.7354	3.31244
C	0.12629	1.33664	1.72222

C	5.27565	-1.88807	-1.59322
C	5.94959	-0.67389	-1.46456
C	5.44824	0.27817	-0.5566
C	4.28526	-0.00508	0.205
C	3.59391	-1.22563	0.10222
C	4.11882	-2.13872	-0.81256
H	6.96349	1.79798	-0.9188
H	6.83809	-0.45406	-2.04542
C	6.06487	1.56866	-0.35402
C	4.34161	2.21858	1.26543
C	5.5503	2.48399	0.50718
H	6.00284	3.45485	0.65974
O	3.77279	0.9305	1.08703
O	3.76042	2.99045	2.03275
O	2.50778	-1.40258	0.92754
C	1.51612	-2.35996	0.72447
H	0.85471	-2.43046	1.57397
H	-0.23453	-1.16588	-0.23437
H	1.67766	-3.14674	0.00256
O	3.62532	-3.40936	-1.10011
C	4.48841	-3.95351	-2.0775
H	4.22762	-4.94377	-2.40675
C	5.47767	-3.08319	-2.39392
H	6.26763	-3.24887	-3.10928

[4]TS-reb$_{A}$

Fe	1.94896	-0.27503	-0.17358
O	0.78626	1.03688	0.00445
S	3.45094	-2.13729	-0.16406
H	2.62342	-3.05513	-0.78198
N	0.96794	-1.05052	-1.73921
C	-0.58659	-2.17043	-3.04361
C	0.22866	-1.47647	-3.8929
H	-1.42132	-2.81113	-3.28832
H	0.19088	-1.43124	-4.97164
N	3.07179	0.81917	-1.39437
C	3.0089	0.85584	-2.7793
C	3.97822	1.80263	-3.28995
C	4.62654	2.33376	-2.21321
H	4.12936	2.01837	-4.33767
H	5.4182	3.06884	-2.20235
N	3.04362	0.3375	1.41189
C	4.05174	1.28419	1.39299
C	2.89645	-0.05	2.73706
C	4.55729	1.48883	2.73243
C	3.84874	0.66309	3.55953
H	5.35184	2.1742	2.98951

H	3.94693	0.54019	4.62837
N	0.83637	-1.44468	1.08709
C	-0.0259	-2.48071	2.97472
H	-0.12949	-2.75258	4.01513
C	0.99226	-1.58743	2.45794
C	-0.1292	-1.90386	-1.69781
C	1.18823	-0.76708	-3.07625
C	4.07086	1.71057	-1.02988
C	1.95346	-0.94535	3.22274
H	1.96233	-1.14801	4.28852
C	-0.7035	-2.43829	-0.55443
H	-1.56673	-3.08333	-0.67301
C	2.14864	0.10768	-3.56412
H	2.21026	0.23546	-4.63951
C	4.51996	1.93582	0.26003
H	5.31869	2.65766	0.3931
C	-0.7953	-2.87124	1.91719
H	-1.66943	-3.50466	1.90395
C	-0.2447	-2.234	0.7385
C	-4.55296	2.73907	-0.25064
C	-5.39217	1.75327	-0.76883
C	-5.10042	0.40662	-0.48175
C	-3.97583	0.07453	0.31785
C	-3.1185	1.04848	0.86281
C	-3.44326	2.36987	0.55105
H	-6.77357	-0.4519	-1.57679
H	-6.25135	2.00344	-1.38084
C	-5.90085	-0.68929	-0.97578
C	-4.41553	-2.33763	0.06755
C	-5.58599	-1.98421	-0.71425
H	-6.1759	-2.81115	-1.08687
O	-3.66958	-1.24885	0.58741
O	-4.00181	-3.47435	0.31287
O	-2.10087	0.61296	1.67472
C	-0.90422	1.34848	1.88952
H	-0.40939	0.93055	2.76078
H	-1.01462	2.42986	1.84836
O	-2.76748	3.51369	0.96913
C	-3.46408	4.60806	0.41104
H	-3.04827	5.5723	0.64418
C	-4.5281	4.18982	-0.31653
H	-5.22523	4.81921	-0.84678
H	0.01799	1.08587	0.84323

^{2}P-OH$_{A}$

Fe	-1.91288	0.25528	-0.38389
O	-0.38886	-0.26595	0.98028

S	-3.61759	0.66614	-1.85872
H	-3.81781	2.00021	-1.55856
N	-0.99859	2.00223	-0.78818
C	-0.38475	4.24228	-0.83071
C	0.30449	3.61625	-1.82893
H	-0.35137	5.28339	-0.5443
H	1.01653	4.04321	-2.51996
N	-0.84117	-0.68795	-1.81185
C	0.04869	-0.08126	-2.6845
C	0.55894	-1.05879	-3.6241
C	-0.03111	-2.25307	-3.32295
H	1.26851	-0.84081	-4.40928
H	0.0987	-3.20755	-3.81258
N	-2.71642	-1.5333	0.11531
C	-2.54601	-2.75719	-0.51792
C	-3.66098	-1.74245	1.10967
C	-3.38837	-3.7551	0.10565
C	-4.07479	-3.13014	1.10859
H	-3.44567	-4.78951	-0.20182
H	-4.80335	-3.55287	1.78547
N	-2.88098	1.16459	1.13218
C	-4.32678	1.54606	2.90559
H	-5.05697	1.3341	3.67337
C	-3.7927	0.56214	1.98632
C	-1.19997	3.23691	-0.18271
C	-0.08062	2.22027	-1.80496
C	-0.90788	-2.02054	-2.19466
C	-4.15114	-0.77821	1.97857
H	-4.88744	-1.09768	2.70889
C	-2.05433	3.47287	0.88465
H	-2.11811	4.48683	1.26473
C	0.4056	1.2592	-2.67805
H	1.12222	1.58556	-3.42435
C	-1.69818	-2.9886	-1.59214
H	-1.66211	-3.99366	-1.99913
C	-3.73928	2.74184	2.60321
H	-3.89236	3.70227	3.07395
C	-2.83917	2.50365	1.49518
C	3.60423	-2.60607	0.91156
C	4.91489	-2.18886	0.6866
C	5.16686	-0.80997	0.56357
C	4.10599	0.12715	0.67242
C	2.77319	-0.26538	0.90137
C	2.5738	-1.64057	1.00965
H	7.31355	-0.98311	0.24016
H	5.72989	-2.8987	0.60229

C	6.49095	-0.27947	0.32622
C	5.64367	2.02691	0.32095
C	6.71759	1.05222	0.2099
H	7.70464	1.45785	0.03056
O	4.34488	1.48482	0.55943
O	5.74365	3.24758	0.23378
O	1.80905	0.72307	0.94015
C	0.56621	0.52555	1.67675
H	0.14504	1.51991	1.7651
H	0.7917	0.07492	2.64869
O	1.34994	-2.29233	1.21627
C	1.63596	-3.68127	1.24267
H	0.77803	-4.31399	1.38653
C	2.95994	-3.9001	1.06887
H	3.44582	-4.86286	1.04573
H	-0.14189	-1.21709	0.95585

^{4}P-OH$_{A}$

Fe	-1.90292	-0.2446	0.47251
O	-0.47218	0.32976	-1.52383
S	-3.27517	-0.6501	2.49959
H	-4.50183	-0.78497	1.87603
N	-1.62302	-2.19097	0.01018
C	-1.97022	-4.35685	-0.74751
C	-0.85812	-4.38165	0.04508
H	-2.43564	-5.17706	-1.27457
H	-0.23025	-5.22464	0.29366
N	-0.21223	-0.2629	1.5611
C	0.55906	-1.37369	1.87658
C	1.62569	-0.9908	2.7718
C	1.50331	0.35308	2.99371
H	2.36475	-1.67171	3.16794
H	2.12197	0.98912	3.61014
N	-1.94341	1.78259	0.60757
C	-1.13044	2.57859	1.40603
C	-2.90935	2.62984	0.07733
C	-1.57891	3.95162	1.3463
C	-2.67363	3.98348	0.52704
H	-1.11915	4.77034	1.88107
H	-3.28423	4.83355	0.25853
N	-3.46251	-0.18	-0.79702
C	-5.29972	0.54697	-2.01025
H	-6.03425	1.2272	-2.41644
C	-4.21549	0.93692	-1.13883
C	-2.45168	-2.99223	-0.7636
C	-0.64733	-3.03365	0.52607
C	0.36221	0.8085	2.2336

C	-3.96271	2.23971	-0.73492
H	-4.64531	3.01045	-1.07641
C	-3.58096	-2.56148	-1.44162
H	-4.14045	-3.29938	-2.00631
C	0.36449	-2.6585	1.39357
H	1.06034	-3.42517	1.71487
C	-0.06149	2.12782	2.1646
H	0.48423	2.86033	2.74947
C	-5.19498	-0.80243	-2.20657
H	-5.8263	-1.44263	-2.80556
C	-4.04464	-1.2541	-1.45878
C	3.54595	2.54737	-0.83734
C	4.79627	2.06959	-0.44945
C	4.99164	0.67691	-0.39618
C	3.9378	-0.20958	-0.73795
C	2.66816	0.24372	-1.14714
C	2.52064	1.62989	-1.17219
H	7.06597	0.74747	0.26228
H	5.60492	2.74186	-0.18604
C	6.2475	0.08243	0.00321
C	5.3441	-2.18503	-0.27443
C	6.41645	-1.26185	0.06142
H	7.3519	-1.71535	0.36185
O	4.11892	-1.58077	-0.68179
O	5.3905	-3.41249	-0.24212
O	1.7123	-0.70804	-1.42539
C	0.52652	-0.38443	-2.22755
H	0.11361	-1.35897	-2.46299
H	0.85027	0.15766	-3.12318
O	1.35734	2.33927	-1.50433
C	1.67553	3.71318	-1.36608
H	0.86327	4.38652	-1.57557
C	2.9625	3.87151	-0.97863
H	3.46054	4.81166	-0.80054
H	-0.22141	1.27298	-1.40399

Path B

$^{2}RC_{B}$

Fe	-2.57036	-0.02129	0.0319
O	-1.16808	0.66848	-0.50793
S	-4.88913	-0.75417	0.90481
H	-4.59558	-0.77246	2.25598
N	-3.11515	1.5901	1.13039
C	-3.22925	3.18414	2.81724
C	-3.86548	3.70571	1.72779
H	-3.08515	3.63507	3.78819

H	-4.34806	4.66723	1.63103
N	-3.6897	0.66976	-1.49827
C	-4.28487	1.91622	-1.59871
C	-4.90467	2.06532	-2.89797
C	-4.68858	0.9033	-3.58086
H	-5.43917	2.94541	-3.22417
H	-5.00813	0.64261	-4.57917
N	-2.29206	-1.74981	-0.95604
C	-2.71309	-2.05633	-2.23634
C	-1.54552	-2.83549	-0.53031
C	-2.22465	-3.36304	-2.62066
C	-1.49679	-3.84136	-1.56863
H	-2.4185	-3.83492	-3.57271
H	-0.97313	-4.78204	-1.48464
N	-1.69748	-0.81675	1.66724
C	-0.38404	-2.16686	3.02518
H	0.22622	-3.00907	3.3132
C	-1.01499	-2.02243	1.73237
C	-2.747	1.87325	2.44355
C	-3.78985	2.71846	0.6739
C	-3.92468	0.03595	-2.71053
C	-0.93906	-2.95776	0.71179
H	-0.33836	-3.84031	0.89288
C	-2.00134	1.03969	3.25836
H	-1.77374	1.39313	4.25855
C	-4.31396	2.87552	-0.59686
H	-4.81183	3.81339	-0.81899
C	-3.4807	-1.2312	-3.04848
H	-3.74036	-1.60473	-4.03343
C	-0.69301	-1.04892	3.74616
H	-0.39789	-0.79864	4.75459
C	-1.5041	-0.20579	2.89463
C	5.9152	2.05091	-0.95844
C	4.52341	1.9809	-0.99203
C	3.90426	0.79771	-0.54933
C	4.68827	-0.28847	-0.08267
C	6.09669	-0.24741	-0.03645
C	6.66683	0.94485	-0.48595
H	1.84163	1.4374	-0.89046
H	3.91934	2.80787	-1.34742
C	2.46953	0.62314	-0.54228
C	2.67645	-1.64332	0.36994
C	1.88807	-0.52475	-0.11235
H	0.81209	-0.63293	-0.11263
O	4.08737	-1.45863	0.35766
O	2.25699	-2.72657	0.78748

O	6.73906	-1.36591	0.43645
C	8.19818	-1.39709	0.53751
H	8.56131	-0.61906	1.21332
H	8.4154	-2.38445	0.94232
H	8.66256	-1.276	-0.44388
O	8.03346	1.24923	-0.54772
C	8.118	2.5585	-1.06444
H	9.11937	2.93721	-1.17178
C	6.88824	3.06725	-1.31999
H	6.67274	4.04616	-1.71875

^4RC_B

Fe	-2.57904	-0.01618	0.03599
O	-1.17032	0.65073	-0.52408
S	-4.86568	-0.78671	0.90197
H	-4.5791	-0.78086	2.25467
N	-3.13106	1.59826	1.11627
C	-3.27164	3.20055	2.79338
C	-3.91961	3.70334	1.70211
H	-3.13395	3.66011	3.76121
H	-4.42	4.65516	1.60014
N	-3.70086	0.64964	-1.50226
C	-4.31489	1.88599	-1.61172
C	-4.93753	2.01643	-2.91147
C	-4.70435	0.85275	-3.58591
H	-5.48524	2.88604	-3.2438
H	-5.02057	0.58001	-4.58206
N	-2.2796	-1.75325	-0.94068
C	-2.69115	-2.07181	-2.22057
C	-1.52601	-2.8289	-0.50351
C	-2.18755	-3.37614	-2.59456
C	-1.46184	-3.84118	-1.53525
H	-2.37111	-3.85549	-3.54492
H	-0.92945	-4.77609	-1.44233
N	-1.69246	-0.78855	1.67614
C	-0.37011	-2.11794	3.04597
H	0.24452	-2.95427	3.34174
C	-1.00474	-1.9908	1.75295
C	-2.76712	1.89592	2.42755
C	-3.82833	2.71089	0.65452
C	-3.92761	0.00292	-2.70969
C	-0.9241	-2.93649	0.74227
H	-0.31917	-3.81403	0.93331
C	-2.00796	1.08044	3.24829
H	-1.78282	1.44638	4.24452
C	-4.3568	2.85157	-0.61632
H	-4.86971	3.78008	-0.84348

C	-3.46674	-1.2605	-3.03913
H	-3.72061	-1.64314	-4.0221
C	-0.68168	-0.9932	3.75506
H	-0.3851	-0.73038	4.75987
C	-1.49942	-0.16378	2.89642
C	5.91948	2.04432	-0.98494
C	4.52812	1.96968	-1.02555
C	3.90985	0.79127	-0.56909
C	4.69432	-0.28555	-0.082
C	6.10231	-0.23965	-0.02819
C	6.67158	0.94758	-0.49187
H	1.84759	1.42005	-0.93083
H	3.92377	2.7896	-1.39649
C	2.47562	0.61253	-0.56752
C	2.68398	-1.63963	0.37903
C	1.89495	-0.53066	-0.12417
H	0.81926	-0.64183	-0.12822
O	4.09424	-1.4509	0.37203
O	2.26539	-2.71789	0.81022
O	6.74506	-1.34909	0.46496
C	8.20373	-1.375	0.57405
H	8.56142	-0.58571	1.2396
H	8.42126	-2.35545	0.99516
H	8.67291	-1.26791	-0.40671
O	8.0376	1.25519	-0.55002
C	8.12134	2.55701	-1.08541
H	9.12221	2.93716	-1.19237
C	6.89162	3.05819	-1.35565
H	6.6756	4.0305	-1.76999

^{2}TS-O$_{B}$

Fe	-1.86765	0.16023	0.29164
O	-0.18365	-0.17767	-0.06998
S	-4.06246	0.77271	1.22373
H	-4.04653	-0.04283	2.33904
N	-1.20233	0.73788	2.12157
C	-0.41003	0.73397	4.30377
C	-0.30061	2.01227	3.83887
H	-0.14265	0.34928	5.27716
H	0.0723	2.88394	4.35671
N	-1.8248	2.06789	-0.34931
C	-1.33808	3.15831	0.35694
C	-1.44621	4.35451	-0.45048
C	-2.01085	3.98878	-1.63988
H	-1.13975	5.3397	-0.13004
H	-2.25614	4.6142	-2.48592
N	-2.66449	-0.38678	-1.46545

C	-2.99663	0.42659	-2.54171
C	-3.03062	-1.67143	-1.83578
C	-3.56521	-0.369	-3.60742
C	-3.58121	-1.66401	-3.1736
H	-3.90457	0.02514	-4.55418
H	-3.934	-2.54146	-3.69525
N	-2.0621	-1.72269	0.99811
C	-2.37338	-4.02039	1.08662
H	-2.62889	-5.01439	0.75045
C	-2.46856	-2.82714	0.27129
C	-0.96368	-0.0632	3.22807
C	-0.79161	2.01325	2.47506
C	-2.2455	2.56315	-1.57574
C	-2.91606	-2.80296	-1.0408
H	-3.21187	-3.7489	-1.48125
C	-1.20278	-1.42692	3.28783
H	-0.9644	-1.9361	4.21578
C	-0.84438	3.12808	1.65363
H	-0.49149	4.0664	2.06932
C	-2.80058	1.79939	-2.59237
H	-3.10013	2.31433	-3.49924
C	-1.90287	-3.63221	2.30736
H	-1.69742	-4.24463	3.17307
C	-1.70103	-2.19906	2.24683
C	4.9247	1.4269	-0.57061
C	3.65649	1.67157	-1.08903
C	2.78095	0.57784	-1.27163
C	3.20759	-0.73872	-0.9344
C	4.48234	-1.01355	-0.40708
C	5.3095	0.10092	-0.24349
H	1.11055	1.72746	-2.05068
H	3.32788	2.67239	-1.34467
C	1.45542	0.73562	-1.78036
C	1.06953	-1.70972	-1.66338
C	0.57391	-0.33771	-1.83997
O	2.37706	-1.83071	-1.13924
O	0.45645	-2.74197	-1.92062
O	4.78423	-2.32702	-0.1406
C	5.96156	-2.66712	0.65975
H	5.9236	-2.17547	1.63598
H	5.89938	-3.74783	0.77848
H	6.8828	-2.38958	0.1442
O	6.6203	0.10297	0.24599
C	7.04421	1.44907	0.22272
H	8.04718	1.61185	0.57642
C	6.07073	2.26234	-0.25328

H	6.13593	3.33224	-0.37476
H	-0.3649	-0.26009	-2.36836

^{4}TS-O$_B$

Fe	-1.85506	0.14621	0.36027
O	-0.20736	-0.01098	-0.18986
S	-4.19593	0.28941	0.98779
H	-4.4638	1.5504	0.49127
N	-1.21055	0.4859	2.21526
C	-0.485	0.19624	4.39975
C	-0.41537	1.53124	4.1292
H	-0.23078	-0.31753	5.31531
H	-0.09322	2.3318	4.77907
N	-1.8802	2.14231	-0.01504
C	-1.4479	3.13863	0.84114
C	-1.53772	4.42489	0.18675
C	-2.01578	4.20087	-1.07455
H	-1.26689	5.36575	0.64344
H	-2.21429	4.92226	-1.85403
N	-2.61569	-0.17055	-1.515
C	-2.88031	0.78041	-2.4921
C	-2.95481	-1.39363	-2.0653
C	-3.38764	0.13061	-3.68221
C	-3.43146	-1.20936	-3.41928
H	-3.67234	0.64558	-4.58824
H	-3.75594	-2.00954	-4.06809
N	-2.05141	-1.79852	0.77014
C	-2.39525	-4.08213	0.55803
H	-2.65529	-5.02168	0.09352
C	-2.46151	-2.79779	-0.10407
C	-0.98717	-0.45401	3.20674
C	-0.87506	1.71515	2.76761
C	-2.22349	2.77569	-1.20314
C	-2.8673	-2.61541	-1.4143
H	-3.14458	-3.50041	-1.97585
C	-1.22702	-1.81333	3.09024
H	-1.00781	-2.43806	3.94922
C	-0.983	2.94063	2.13586
H	-0.68263	3.81942	2.69666
C	-2.68831	2.14638	-2.35008
H	-2.92643	2.77546	-3.20149
C	-1.94296	-3.85986	1.8259
H	-1.76071	-4.58067	2.60931
C	-1.72113	-2.43602	1.95489
C	4.99403	1.45983	-0.63562
C	3.74954	1.71321	-1.20593
C	2.83835	0.64063	-1.34177

C	3.21497	-0.66462	-0.91327
C	4.46302	-0.94848	-0.33233
C	5.32317	0.14739	-0.20994
H	1.22737	1.79294	-2.2332
H	3.46334	2.70676	-1.53131
C	1.5322	0.80993	-1.89205
C	1.07654	-1.61478	-1.66088
C	0.60195	-0.23512	-1.87997
O	2.35782	-1.74463	-1.08977
O	0.45708	-2.64133	-1.92809
O	4.69681	-2.24809	0.04647
C	5.99616	-2.64192	0.59069
H	6.20857	-2.11406	1.52309
H	5.89472	-3.71085	0.77242
H	6.7967	-2.45227	-0.12874
O	6.61355	0.14278	0.33347
C	7.08179	1.47031	0.23865
H	8.07506	1.62636	0.62163
C	6.15425	2.27918	-0.32819
H	6.25841	3.33649	-0.51518
H	-0.30795	-0.16448	-2.4588

^{2}INT$_B$

Fe	-1.88127	0.36667	0.32999
O	0.04038	-0.54693	-0.25219
S	-3.86424	1.25592	1.05117
H	-3.35625	2.04216	2.06815
N	-0.91331	2.13324	0.41953
C	0.27357	3.98323	1.1684
C	-0.15973	4.268	-0.09697
H	0.85174	4.60729	1.83458
H	-0.00664	5.17124	-0.67021
N	-2.34737	0.70595	-1.60904
C	-2.16114	1.89943	-2.2994
C	-2.75968	1.80669	-3.61326
C	-3.31805	0.56371	-3.71586
H	-2.7514	2.59897	-4.3479
H	-3.85748	0.13852	-4.54979
N	-2.69461	-1.46478	0.16528
C	-3.35191	-2.01479	-0.92744
C	-2.80475	-2.3988	1.18505
C	-3.86498	-3.32534	-0.58801
C	-3.53115	-3.56026	0.71394
H	-4.40675	-3.96645	-1.26786
H	-3.74422	-4.4334	1.31323
N	-1.27059	-0.03179	2.19844
C	-0.99697	-1.07207	4.2544

H	-1.08715	-1.83038	5.01855
C	-1.57335	-1.16971	2.92882
C	-0.20324	2.6574	1.49315
C	-0.90184	3.11606	-0.56311
C	-3.07344	-0.11738	-2.46422
C	-2.29116	-2.26182	2.46477
H	-2.45062	-3.0828	3.15565
C	-0.00715	2.02069	2.70984
H	0.56723	2.54584	3.46553
C	-1.48681	3.01213	-1.81806
H	-1.40802	3.87054	-2.47702
C	-3.52263	-1.39342	-2.15544
H	-4.07132	-1.93267	-2.91997
C	-0.3404	0.12299	4.32025
H	0.21224	0.54004	5.14961
C	-0.51255	0.77249	3.0378
C	4.39968	1.23926	-0.51833
C	3.0501	1.27534	-0.8856
C	2.36645	0.06107	-1.00575
C	3.02376	-1.1638	-0.7607
C	4.37631	-1.2384	-0.38286
C	5.02651	-0.00381	-0.27418
H	0.49965	0.90014	-1.8134
H	2.53163	2.21176	-1.05622
C	0.93875	0.02541	-1.35192
C	1.06786	-2.53667	-1.37859
C	0.2724	-1.28638	-1.54253
H	-0.6103	-1.38237	-2.1598
O	2.37203	-2.40095	-0.90945
O	0.6407	-3.65453	-1.64152
O	4.90403	-2.48731	-0.17932
C	6.28486	-2.64947	0.2809
H	6.43296	-2.16496	1.24892
H	6.4081	-3.72739	0.37198
H	6.99072	-2.24301	-0.44651
O	6.3643	0.2084	0.07671
C	6.55751	1.60406	0.04976
H	7.55226	1.92735	0.30166
C	5.41701	2.25022	-0.29792
H	5.29116	3.31733	-0.39255

^{4}INT$_{B}$

Fe	-2.7482	0.01229	0.10532
O	-0.30141	-0.76702	-0.68043
S	-5.00069	0.90651	0.94703
H	-4.53606	1.69996	1.97951
N	-2.50697	1.99237	-0.20298

C	-1.78664	4.19369	-0.00599
C	-2.3763	4.07201	-1.2308
H	-1.33862	5.07085	0.43735
H	-2.50766	4.8302	-1.98869
N	-3.76097	-0.11801	-1.63321
C	-3.89586	0.86842	-2.59583
C	-4.59864	0.34483	-3.74758
C	-4.89153	-0.96057	-3.4785
H	-4.8346	0.91659	-4.63295
H	-5.41398	-1.67271	-4.10014
N	-3.25691	-1.88938	0.53852
C	-3.9239	-2.77926	-0.28132
C	-2.92395	-2.59818	1.67929
C	-4.01191	-4.07468	0.35778
C	-3.39257	-3.96295	1.56973
H	-4.48916	-4.94154	-0.07503
H	-3.26105	-4.72008	2.32868
N	-1.97288	0.22355	1.95726
C	-1.10018	-0.25517	4.05439
H	-0.84752	-0.83167	4.9321
C	-1.80653	-0.77598	2.90439
C	-1.85375	2.89776	0.6317
C	-2.81779	2.70121	-1.35999
C	-4.36398	-1.2512	-2.16276
C	-2.25129	-2.08237	2.77761
H	-2.05658	-2.75722	3.60442
C	-1.32646	2.59858	1.87485
H	-0.83166	3.39991	2.41329
C	-3.44473	2.17459	-2.4749
H	-3.61442	2.84092	-3.31376
C	-4.44342	-2.48219	-1.53495
H	-4.94773	-3.28178	-2.06717
C	-0.83576	1.06003	3.79896
H	-0.32352	1.77521	4.42557
C	-1.37996	1.35399	2.49117
C	3.382	2.41069	0.65393
C	2.31155	1.8956	-0.07554
C	2.15146	0.49814	-0.13282
C	3.06593	-0.3564	0.53558
C	4.15718	0.13467	1.2782
C	4.27253	1.52527	1.31433
H	0.36747	0.50704	-1.37836
H	1.6098	2.54311	-0.58947
C	1.07601	-0.13347	-0.86108
C	1.8666	-2.3727	-0.23284
C	0.93791	-1.48243	-0.9101

H	0.13504	-1.96332	-1.45347
O	2.91935	-1.73587	0.47929
O	1.82809	-3.60277	-0.21912
O	4.9626	-0.78876	1.90193
C	6.28009	-0.39255	2.40258
H	6.19196	0.3276	3.21773
H	6.72217	-1.32371	2.75461
H	6.88746	0.03655	1.60036
O	5.24687	2.26259	1.98947
C	4.96042	3.62661	1.74011
H	5.63741	4.32981	2.20508
C	3.86232	3.74812	0.9528
H	3.42791	4.67531	0.61345

^{2}P-epo$_{B}$

Fe	-2.7482	0.01229	0.10532
O	-0.04974	-0.80945	-0.74407
S	-5.00069	0.90651	0.94703
H	-4.53606	1.69996	1.97951
N	-2.50697	1.99237	-0.20298
C	-1.78664	4.19369	-0.00599
C	-2.3763	4.07201	-1.2308
H	-1.33862	5.07085	0.43735
H	-2.50766	4.8302	-1.98869
N	-3.76097	-0.11801	-1.63321
C	-3.89586	0.86842	-2.59583
C	-4.59864	0.34483	-3.74758
C	-4.89153	-0.96057	-3.4785
H	-4.8346	0.91659	-4.63295
H	-5.41398	-1.67271	-4.10014
N	-3.25691	-1.88938	0.53852
C	-3.9239	-2.77926	-0.28132
C	-2.92395	-2.59818	1.67929
C	-4.01191	-4.07468	0.35778
C	-3.39257	-3.96295	1.56973
H	-4.48916	-4.94154	-0.07503
H	-3.26105	-4.72008	2.32868
N	-1.97288	0.22355	1.95726
C	-1.10018	-0.25517	4.05439
H	-0.84752	-0.83167	4.9321
C	-1.80653	-0.77598	2.90439
C	-1.85375	2.89776	0.6317
C	-2.81779	2.70121	-1.35999
C	-4.36398	-1.2512	-2.16276
C	-2.25129	-2.08237	2.77761
H	-2.05658	-2.75722	3.60442
C	-1.32646	2.59858	1.87485

H	-0.83166	3.39991	2.41329
C	-3.44473	2.17459	-2.4749
H	-3.61442	2.84092	-3.31376
C	-4.44342	-2.48219	-1.53495
H	-4.94773	-3.28178	-2.06717
C	-0.83576	1.06003	3.79896
H	-0.32352	1.77521	4.42557
C	-1.37996	1.35399	2.49117
C	3.5315	2.39074	0.60731
C	2.46104	1.87565	-0.12216
C	2.30096	0.47819	-0.17943
C	3.21543	-0.37635	0.48896
C	4.30668	0.11472	1.23159
C	4.42203	1.50533	1.26771
H	0.51697	0.4871	-1.42498
H	1.75929	2.52317	-0.63609
C	1.2255	-0.15341	-0.90769
C	2.0161	-2.39264	-0.27946
C	1.08741	-1.50237	-0.95672
H	0.28454	-1.98327	-1.50009
O	3.06885	-1.75582	0.43267
O	1.97759	-3.62271	-0.26573
O	5.1121	-0.8087	1.85531
C	6.42959	-0.41249	2.35596
H	6.34146	0.30766	3.17111
H	6.87167	-1.34366	2.70799
H	7.03696	0.0166	1.55374
O	5.39637	2.24264	1.94285
C	5.10992	3.60667	1.6935
H	5.78691	4.30987	2.15846
C	4.01182	3.72817	0.90618
H	3.5774	4.65536	0.56683

^{4}P-epo$_B$

Fe	-1.67907	0.23673	0.63175
O	-0.45784	-0.05669	-1.60728
S	-2.97797	0.56342	2.59538
H	-1.95559	0.55229	3.52607
N	-1.32667	2.2574	0.21008
C	-0.49663	4.41201	0.31369
C	-1.65575	4.458	-0.41438
H	0.15846	5.23132	0.57371
H	-2.12629	5.32165	-0.86227
N	-3.32877	0.29495	-0.5102
C	-3.91825	1.43245	-1.04759
C	-5.16123	1.06012	-1.69384
C	-5.30473	-0.29157	-1.55686

H	-5.82147	1.75428	-2.19337
H	-6.10442	-0.91872	-1.92346
N	-1.74375	-1.85217	0.59071
C	-2.7724	-2.62666	0.08757
C	-0.87022	-2.71082	1.23245
C	-2.51558	-4.02233	0.38764
C	-1.34317	-4.07437	1.0903
H	-3.15411	-4.84388	0.09718
H	-0.83956	-4.94615	1.48158
N	0.0776	0.15286	1.59783
C	1.87183	-0.60435	2.84388
H	2.52753	-1.29929	3.34771
C	0.67469	-0.9877	2.12565
C	-0.3011	3.03261	0.71675
C	-2.18242	3.10751	-0.4638
C	-4.15229	-0.77815	-0.82467
C	0.24819	-2.30136	1.95004
H	0.85276	-3.07498	2.41082
C	0.71868	2.55178	1.53284
H	1.45651	3.26741	1.88091
C	-3.39005	2.71991	-1.03702
H	-3.98093	3.49252	-1.51845
C	-3.8959	-2.12022	-0.55735
H	-4.64169	-2.83504	-0.88932
C	2.00115	0.75215	2.73257
H	2.783	1.38285	3.13039
C	0.88761	1.23148	1.9405
C	3.98922	1.49907	-1.13237
C	2.76697	1.50595	-1.81361
C	2.02618	0.32102	-1.85811
C	2.50984	-0.85185	-1.23901
C	3.7262	-0.8935	-0.53573
C	4.43278	0.31345	-0.50531
H	0.47163	1.05379	-3.22344
H	2.38483	2.40582	-2.28161
C	0.70628	0.27285	-2.5093
C	0.67199	-2.26111	-2.08706
C	0.0095	-1.04015	-2.62708
H	-0.69981	-1.23641	-3.42114
O	1.82094	-2.07406	-1.32268
O	0.26841	-3.40024	-2.29138
O	4.07927	-2.08407	0.04868
C	5.43513	-2.28071	0.56725
H	5.6334	-1.61609	1.40991
H	5.44934	-3.32167	0.88653
H	6.17934	-2.11413	-0.21625

O	5.65752	0.54976	0.12893
C	5.96895	1.90198	-0.11709
H	6.89713	2.23731	0.3114
C	5.00486	2.49936	-0.86095
H	4.99616	3.52747	-1.18744

Path C

^{2}RC$_{C}$

Fe	-2.74512	-0.00678	0.10705
O	-1.31564	-0.35732	-0.64099
S	-4.92635	0.90554	1.14444
H	-4.41703	1.31708	2.36224
N	-2.308	1.95939	0.31617
C	-1.3664	3.9499	1.0603
C	-1.92901	4.20202	-0.15737
H	-0.84275	4.63502	1.71075
H	-1.95787	5.13481	-0.70106
N	-3.71523	0.41319	-1.61179
C	-3.72438	1.61999	-2.29099
C	-4.43628	1.47833	-3.54327
C	-4.8592	0.18314	-3.61937
H	-4.58811	2.27653	-4.25479
H	-5.42556	-0.29249	-4.4065
N	-3.4456	-1.89032	0.0384
C	-4.17054	-2.47215	-0.98461
C	-3.2229	-2.89366	0.96561
C	-4.40703	-3.86972	-0.69418
C	-3.82021	-4.13056	0.51105
H	-4.95337	-4.54588	-1.33517
H	-3.79011	-5.0631	1.05511
N	-2.01602	-0.346	1.9575
C	-1.26253	-1.42129	3.87152
H	-1.09637	-2.22195	4.57709
C	-1.97753	-1.56224	2.62207
C	-1.58789	2.55106	1.35224
C	-2.50707	2.96272	-0.62769
C	-4.40398	-0.48262	-2.41786
C	-2.54061	-2.74292	2.16404
H	-2.44012	-3.62031	2.79418
C	-1.13361	1.89472	2.4818
H	-0.57593	2.47722	3.20738
C	-3.15184	2.8006	-1.8407
H	-3.22965	3.66976	-2.48495
C	-4.61868	-1.81838	-2.12531
H	-5.17901	-2.40348	-2.84679
C	-0.86293	-0.11827	3.95815

H	-0.30376	0.36082	4.74829
C	-1.33249	0.54825	2.76319
C	3.96463	-1.44114	-0.94064
C	5.18386	-2.09615	-1.10756
C	6.36591	-1.40377	-0.78322
C	6.30702	-0.06934	-0.30389
C	5.09092	0.61827	-0.12714
C	3.945	-0.10942	-0.45186
H	7.73884	-3.01496	-1.2864
H	5.23616	-3.11522	-1.47453
C	7.67682	-1.99407	-0.92062
C	8.76554	0.0565	-0.11833
C	8.80729	-1.31163	-0.60838
H	9.79308	-1.74673	-0.70847
O	7.46821	0.62332	0.01129
O	9.72977	0.75725	0.18813
O	5.1526	1.89894	0.37034
C	4.01236	2.8026	0.20138
H	3.14571	2.45426	0.76524
H	4.3686	3.7568	0.58736
H	3.74671	2.8971	-0.85584
O	2.62419	0.32926	-0.34761
C	1.81057	-0.7444	-0.78456
H	0.74644	-0.55426	-0.75824
C	2.57394	-1.80731	-1.14239
H	2.20726	-2.75313	-1.50916

^{4}RC$_C$

Fe	-2.7482	0.01229	0.10532
O	-1.29661	-0.41809	-0.55892
S	-5.00069	0.90651	0.94703
H	-4.53606	1.69996	1.97951
N	-2.50697	1.99237	-0.20298
C	-1.78664	4.19369	-0.00599
C	-2.3763	4.07201	-1.2308
H	-1.33862	5.07085	0.43735
H	-2.50766	4.8302	-1.98869
N	-3.76097	-0.11801	-1.63321
C	-3.89586	0.86842	-2.59583
C	-4.59864	0.34483	-3.74758
C	-4.89153	-0.96057	-3.4785
H	-4.8346	0.91659	-4.63295
H	-5.41398	-1.67271	-4.10014
N	-3.25691	-1.88938	0.53852
C	-3.9239	-2.77926	-0.28132
C	-2.92395	-2.59818	1.67929
C	-4.01191	-4.07468	0.35778

C	-3.39257	-3.96295	1.56973
H	-4.48916	-4.94154	-0.07503
H	-3.26105	-4.72008	2.32868
N	-1.97288	0.22355	1.95726
C	-1.10018	-0.25517	4.05439
H	-0.84752	-0.83167	4.9321
C	-1.80653	-0.77598	2.90439
C	-1.85375	2.89776	0.6317
C	-2.81779	2.70121	-1.35999
C	-4.36398	-1.2512	-2.16276
C	-2.25129	-2.08237	2.77761
H	-2.05658	-2.75722	3.60442
C	-1.32646	2.59858	1.87485
H	-0.83166	3.39991	2.41329
C	-3.44473	2.17459	-2.4749
H	-3.61442	2.84092	-3.31376
C	-4.44342	-2.48219	-1.53495
H	-4.94773	-3.28178	-2.06717
C	-0.83576	1.06003	3.79896
H	-0.32352	1.77521	4.42557
C	-1.37996	1.35399	2.49117
C	3.99405	-1.49327	-0.87557
C	5.21894	-2.13454	-1.05368
C	6.39626	-1.42484	-0.75031
C	6.32665	-0.08808	-0.2797
C	5.10447	0.58603	-0.09177
C	3.96353	-0.15784	-0.39722
H	7.78303	-3.02206	-1.26007
H	5.27907	-3.15553	-1.41397
C	7.71274	-1.99947	-0.90061
C	8.78547	0.07029	-0.12649
C	8.83835	-1.30056	-0.60784
H	9.82823	-1.72383	-0.71775
O	7.48282	0.62145	0.01596
O	9.74457	0.78518	0.16298
O	5.161	1.8721	0.39123
C	3.98672	2.74161	0.2994
H	3.16988	2.36786	0.91904
H	4.33985	3.70527	0.66421
H	3.64826	2.82897	-0.73712
O	2.63825	0.26551	-0.28242
C	1.83346	-0.82162	-0.70107
H	0.76763	-0.6434	-0.66595
C	2.60581	-1.87811	-1.0585
H	2.24696	-2.83136	-1.41359

^{2}TS-O$_{C}$

Fe	-2.74512	-0.00678	0.10705
O	-1.31564	-0.35732	-0.64099
S	-4.92635	0.90554	1.14444
H	-4.41703	1.31708	2.36224
N	-2.308	1.95939	0.31617
C	-1.3664	3.9499	1.0603
C	-1.92901	4.20202	-0.15737
H	-0.84275	4.63502	1.71075
H	-1.95787	5.13481	-0.70106
N	-3.71523	0.41319	-1.61179
C	-3.72438	1.61999	-2.29099
C	-4.43628	1.47833	-3.54327
C	-4.8592	0.18314	-3.61937
H	-4.58811	2.27653	-4.25479
H	-5.42556	-0.29249	-4.4065
N	-3.4456	-1.89032	0.0384
C	-4.17054	-2.47215	-0.98461
C	-3.2229	-2.89366	0.96561
C	-4.40703	-3.86972	-0.69418
C	-3.82021	-4.13056	0.51105
H	-4.95337	-4.54588	-1.33517
H	-3.79011	-5.0631	1.05511
N	-2.01602	-0.346	1.9575
C	-1.26253	-1.42129	3.87152
H	-1.09637	-2.22195	4.57709
C	-1.97753	-1.56224	2.62207
C	-1.58789	2.55106	1.35224
C	-2.50707	2.96272	-0.62769
C	-4.40398	-0.48262	-2.41786
C	-2.54061	-2.74292	2.16404
H	-2.44012	-3.62031	2.79418
C	-1.13361	1.89472	2.4818
H	-0.57593	2.47722	3.20738
C	-3.15184	2.8006	-1.8407
H	-3.22965	3.66976	-2.48495
C	-4.61868	-1.81838	-2.12531
H	-5.17901	-2.40348	-2.84679
C	-0.86293	-0.11827	3.95815
H	-0.30376	0.36082	4.74829
C	-1.33249	0.54825	2.76319
C	1.60581	-2.65153	0.26255
C	2.00103	-3.77211	0.99161
C	2.43719	-3.58874	2.31749
C	2.4751	-2.28942	2.88692
C	2.08421	-1.13932	2.17458
C	1.65032	-1.36929	0.86806

H	2.84333	-5.68543	2.7334
H	1.97577	-4.76758	0.56228
C	2.86639	-4.68451	3.15468
C	3.33631	-3.16654	5.02785
C	3.28888	-4.49069	4.42948
H	3.61198	-5.30629	5.06314
O	2.91085	-2.09873	4.19123
O	3.70422	-2.88829	6.16886
O	2.13566	0.06508	2.83654
C	2.14393	1.31608	2.07483
H	1.19983	1.46389	1.54833
H	2.29226	2.08717	2.82968
H	2.96879	1.32612	1.35599
O	1.19214	-0.41053	-0.03675
C	0.48657	-1.18016	-1.14165
H	0.10911	-0.56355	-1.94572
C	1.09734	-2.43558	-1.08044
H	0.93152	-3.19149	-1.83188

^{4}TS-O$_{C}$

Fe	-2.74512	-0.00678	0.10705
O	-1.31564	-0.35732	-0.64099
S	-4.92635	0.90554	1.14444
H	-4.41703	1.31708	2.36224
N	-2.308	1.95939	0.31617
C	-1.3664	3.9499	1.0603
C	-1.92901	4.20202	-0.15737
H	-0.84275	4.63502	1.71075
H	-1.95787	5.13481	-0.70106
N	-3.71523	0.41319	-1.61179
C	-3.72438	1.61999	-2.29099
C	-4.43628	1.47833	-3.54327
C	-4.8592	0.18314	-3.61937
H	-4.58811	2.27653	-4.25479
H	-5.42556	-0.29249	-4.4065
N	-3.4456	-1.89032	0.0384
C	-4.17054	-2.47215	-0.98461
C	-3.2229	-2.89366	0.96561
C	-4.40703	-3.86972	-0.69418
C	-3.82021	-4.13056	0.51105
H	-4.95337	-4.54588	-1.33517
H	-3.79011	-5.0631	1.05511
N	-2.01602	-0.346	1.9575
C	-1.26253	-1.42129	3.87152
H	-1.09637	-2.22195	4.57709
C	-1.97753	-1.56224	2.62207
C	-1.58789	2.55106	1.35224

C	-2.50707	2.96272	-0.62769
C	-4.40398	-0.48262	-2.41786
C	-2.54061	-2.74292	2.16404
H	-2.44012	-3.62031	2.79418
C	-1.13361	1.89472	2.4818
H	-0.57593	2.47722	3.20738
C	-3.15184	2.8006	-1.8407
H	-3.22965	3.66976	-2.48495
C	-4.61868	-1.81838	-2.12531
H	-5.17901	-2.40348	-2.84679
C	-0.86293	-0.11827	3.95815
H	-0.30376	0.36082	4.74829
C	-1.33249	0.54825	2.76319
C	1.60581	-2.65153	0.26255
C	2.00103	-3.77211	0.99161
C	2.43719	-3.58874	2.31749
C	2.4751	-2.28942	2.88692
C	2.08421	-1.13932	2.17458
C	1.65032	-1.36929	0.86806
H	2.84333	-5.68543	2.7334
H	1.97577	-4.76758	0.56228
C	2.86639	-4.68451	3.15468
C	3.33631	-3.16654	5.02785
C	3.28888	-4.49069	4.42948
H	3.61198	-5.30629	5.06314
O	2.91085	-2.09873	4.19123
O	3.70422	-2.88829	6.16886
O	2.13566	0.06508	2.83654
C	2.14393	1.31608	2.07483
H	1.19983	1.46389	1.54833
H	2.29226	2.08717	2.82968
H	2.96879	1.32612	1.35599
O	1.19214	-0.41053	-0.03675
C	0.48657	-1.18016	-1.14165
H	0.10911	-0.56355	-1.94572
C	1.09734	-2.43558	-1.08044
H	0.93152	-3.19149	-1.83188

^{2}INT$_C$

Fe	-2.13249	-0.2	0.27259
O	-0.03118	0.26841	-0.21576
S	-4.26832	-0.68164	0.93717
H	-4.93308	0.18923	0.09414
N	-1.62435	-2.1507	0.24098
C	-1.42781	-4.37056	-0.41348
C	-0.99122	-4.29216	0.87846
H	-1.48666	-5.24233	-1.04917

H	-0.62386	-5.08728	1.51103
N	-1.50925	-0.096	2.17732
C	-1.01597	-1.12168	2.9679
C	-0.74657	-0.63062	4.30407
C	-1.08504	0.69103	4.31912
H	-0.35467	-1.23235	5.11113
H	-1.02371	1.38966	5.14089
N	-2.47053	1.78267	0.23645
C	-2.38497	2.64077	1.3242
C	-2.91856	2.55786	-0.82743
C	-2.77793	3.97678	0.92889
C	-3.10242	3.92692	-0.39697
H	-2.79763	4.83032	1.59097
H	-3.44215	4.73011	-1.0347
N	-2.59928	-0.27514	-1.69396
C	-3.39249	0.25483	-3.81329
H	-3.76609	0.85841	-4.62783
C	-3.05094	0.76355	-2.50406
C	-1.82669	-3.03607	-0.8101
C	-1.12396	-2.91087	1.28917
C	-1.55914	1.02435	2.99022
C	-3.18031	2.08748	-2.10672
H	-3.54032	2.80018	-2.84106
C	-2.31466	-2.70189	-2.06555
H	-2.42437	-3.50438	-2.78746
C	-0.8262	-2.43115	2.55552
H	-0.44092	-3.13727	3.28329
C	-1.9705	2.28933	2.60002
H	-1.95483	3.07197	3.35114
C	-3.16496	-1.09295	-3.79304
H	-3.31428	-1.80812	-4.5891
C	-2.67457	-1.42502	-2.47353
C	2.23174	-0.43087	-1.01062
C	3.14915	-1.44457	-0.8023
C	4.48217	-1.08901	-0.49697
C	4.84004	0.2755	-0.39399
C	3.91327	1.32758	-0.59819
C	2.62321	0.92626	-0.92504
H	5.256	-3.11629	-0.3329
H	2.8636	-2.48896	-0.85851
C	5.51858	-2.06504	-0.26106
C	7.17925	-0.3041	0.15264
C	6.78976	-1.69895	0.04301
H	7.57392	-2.4231	0.2206
O	6.1316	0.64473	-0.08323
O	8.29178	0.13831	0.4229

O	4.38838	2.61036	-0.50482
C	3.46934	3.713	-0.19258
H	2.90901	3.49633	0.72168
H	4.12466	4.56917	-0.04151
H	2.77796	3.89246	-1.01652
O	1.5582	1.79743	-1.24752
C	0.40125	0.97832	-1.43551
H	-0.36172	1.4469	-2.03456
C	0.7943	-0.44751	-1.33308
H	0.26757	-1.27128	-1.78986

^{4}INT$_C$

Fe	-2.1285	0.04001	0.25469
O	-0.5208	-0.58932	-0.51457
S	-4.12955	0.85519	1.29559
H	-4.99279	-0.09372	0.78267
N	-3.13872	-1.54095	-0.47184
C	-4.28051	-2.96931	-1.89244
C	-4.03737	-3.65808	-0.7365
H	-4.79197	-3.31091	-2.78051
H	-4.30724	-4.67553	-0.49399
N	-1.68488	-1.01349	1.91387
C	-2.0618	-2.31324	2.20425
C	-1.51728	-2.70293	3.48781
C	-0.81254	-1.63631	3.96622
H	-1.6631	-3.67054	3.94517
H	-0.26736	-1.55366	4.89491
N	-1.25925	1.66536	1.03807
C	-0.56793	1.73204	2.23875
C	-1.18908	2.93183	0.4741
C	-0.06454	3.07093	2.43369
C	-0.45392	3.81149	1.35224
H	0.50848	3.38804	3.29246
H	-0.26406	4.8558	1.15152
N	-2.57448	1.09154	-1.41556
C	-2.85451	2.81806	-2.9337
H	-2.79541	3.81294	-3.35026
C	-2.33649	2.44211	-1.63853
C	-3.71733	-1.64955	-1.73106
C	-3.3125	-2.77348	0.14492
C	-0.92671	-0.57642	2.98721
C	-1.69747	3.30157	-0.76075
H	-1.57234	4.33389	-1.06732
C	-3.76512	-0.65267	-2.69256
H	-4.26703	-0.8801	-3.62645
C	-2.82934	-3.1318	1.3932
H	-3.04641	-4.13306	1.74789

C	-0.39376	0.69174	3.13702
H	0.18099	0.89444	4.03344
C	-3.41332	1.69966	-3.48643
H	-3.90509	1.59962	-4.44303
C	-3.25562	0.62583	-2.53207
C	2.54951	-0.99523	-1.57445
C	3.6898	-1.7947	-1.78058
C	4.90179	-1.42179	-1.1773
C	4.96026	-0.24763	-0.37247
C	3.83085	0.56611	-0.13203
C	2.64428	0.17143	-0.73797
H	6.0919	-3.07093	-1.9447
H	3.641	-2.68766	-2.39407
C	6.12215	-2.17224	-1.33549
C	7.36938	-0.59033	0.06691
C	7.28556	-1.78642	-0.74876
H	8.20432	-2.34653	-0.86227
O	6.14615	0.14172	0.21215
O	8.36362	-0.14314	0.63469
O	3.9598	1.64056	0.72813
C	3.43344	2.93825	0.28141
H	2.34965	2.89845	0.16292
H	3.70942	3.63311	1.07378
H	3.91055	3.23669	-0.6587
O	1.44293	0.82124	-0.62526
C	0.38314	-0.01304	-1.38895
H	-0.08081	0.7178	-2.05914
C	1.22497	-1.08222	-2.02432
H	0.7908	-1.85871	-2.63675

^{2}P-epo$_{C}$

Fe	-2.13536	-0.1851	0.26205
O	-0.02578	0.26346	-0.19729
S	-4.30239	-0.64547	0.83552
H	-4.45943	0.34586	1.78603
N	-2.44457	1.80231	0.18867
C	-2.69614	4.03688	0.76947
C	-3.10025	3.91117	-0.52925
H	-2.67276	4.92765	1.38033
H	-3.4718	4.67894	-1.19244
N	-2.55228	-0.34	-1.71229
C	-3.05972	0.67086	-2.52159
C	-3.36712	0.14362	-3.83358
C	-3.05763	-1.1871	-3.81644
H	-3.77288	0.72584	-4.64828
H	-3.16179	-1.90895	-4.61355
N	-1.65747	-2.14381	0.27137

C	-1.76842	-3.05675	-0.77026
C	-1.26937	-2.87837	1.38256
C	-1.42045	-4.38225	-0.30433
C	-1.11643	-4.27265	1.02277
H	-1.42641	-5.27027	-0.91977
H	-0.82119	-5.05339	1.70883
N	-1.56403	-0.00184	2.17523
C	-0.88426	-0.51358	4.33441
H	-0.55975	-1.11399	5.17191
C	-1.17431	-1.03443	3.01374
C	-2.29376	2.722	1.219
C	-2.94467	2.51815	-0.89177
C	-2.56151	-1.4915	-2.49262
C	-1.0465	-2.36679	2.65173
H	-0.73816	-3.06466	3.42304
C	-1.85276	2.42348	2.49912
H	-1.78067	3.23983	3.20978
C	-3.23442	1.99489	-2.14419
H	-3.63025	2.67703	-2.88921
C	-2.17741	-2.75331	-2.06134
H	-2.23383	-3.56761	-2.77587
C	-1.09319	0.83463	4.28944
H	-0.97595	1.55841	5.08288
C	-1.51926	1.15293	2.94271
C	2.23523	-0.45916	-0.9772
C	3.14675	-1.47366	-0.7484
C	4.48155	-1.11946	-0.44923
C	4.84707	0.24463	-0.37372
C	3.92628	1.29789	-0.59886
C	2.63385	0.89722	-0.91716
H	5.24399	-3.14733	-0.24438
H	2.85546	-2.51734	-0.78463
C	5.51257	-2.09636	-0.19396
C	7.18342	-0.3368	0.18293
C	6.78597	-1.73137	0.10199
H	7.56614	-2.45619	0.2938
O	6.14094	0.61288	-0.07113
O	8.29876	0.10467	0.44311
O	4.41068	2.57848	-0.52898
C	3.49633	3.6994	-0.27578
H	2.91067	3.51877	0.63027
H	4.15708	4.55337	-0.13625
H	2.82787	3.85909	-1.12246
O	1.5722	1.76727	-1.2561
C	0.41298	0.95013	-1.4308
H	-0.3484	1.41115	-2.03769

C	0.79811	-0.47489	-1.30205
H	0.26833	-1.30348	-1.74578

^{4}P-epo$_{C}$

Fe	-1.918	-0.15399	0.56334
O	-0.56703	0.12102	-1.45701
S	-3.31306	-0.49619	2.45761
H	-2.65185	0.32225	3.35428
N	-2.96328	1.48489	-0.22262
C	-3.79422	3.6189	-0.53325
C	-4.60187	2.76293	-1.23271
H	-3.88169	4.69231	-0.44468
H	-5.47408	3.00528	-1.82282
N	-3.0553	-1.40018	-0.53511
C	-4.18321	-1.05086	-1.26585
C	-4.78498	-2.25413	-1.80794
C	-4.0152	-3.31127	-1.41207
H	-5.67709	-2.27029	-2.41718
H	-4.15602	-4.35907	-1.63496
N	-0.59356	-1.73645	0.95454
C	-0.79529	-3.0646	0.63446
C	0.53886	-1.67357	1.7419
C	0.27022	-3.86398	1.21183
C	1.09282	-3.00646	1.89352
H	0.35651	-4.9376	1.12216
H	1.9782	-3.24694	2.46467
N	-0.67473	1.11011	1.50929
C	1.02461	1.95758	2.82766
H	1.91252	1.97388	3.44299
C	0.46251	0.76261	2.23069
C	-2.77396	2.81152	0.11099
C	-4.08572	1.42187	-1.02397
C	-2.92291	-2.78034	-0.61913
C	1.02256	-0.50909	2.33329
H	1.92068	-0.59564	2.93637
C	-1.78204	3.2699	0.97369
H	-1.74575	4.33763	1.16493
C	-4.64986	0.24087	-1.49987
H	-5.54539	0.3305	-2.10648
C	-1.88441	-3.54383	-0.09026
H	-1.93446	-4.61495	-0.25864
C	0.2382	3.01234	2.45294
H	0.35708	4.05561	2.70809
C	-0.82257	2.48839	1.61505
C	1.82018	-0.55163	-1.81256
C	2.69574	-1.55603	-1.44353
C	3.95435	-1.18893	-0.91678

C	4.27914	0.17866	-0.75996
C	3.39364	1.22147	-1.12557
C	2.18133	0.80843	-1.66742
H	4.70186	-3.20944	-0.61426
H	2.42838	-2.60284	-1.53069
C	4.94105	-2.15614	-0.50198
C	6.49235	-0.38162	0.18613
C	6.13874	-1.77967	0.0147
H	6.88643	-2.49723	0.32607
O	5.49561	0.55843	-0.23056
O	7.53749	0.06933	0.64664
O	3.83519	2.51052	-0.969
C	2.86598	3.59308	-0.7436
H	2.23082	3.36068	0.11627
H	3.48623	4.46308	-0.5335
H	2.25097	3.75847	-1.62872
O	1.18369	1.6716	-2.17075
C	0.07474	0.84434	-2.54852
H	-0.55573	1.31464	-3.28634
C	0.45485	-0.58405	-2.37115
H	0.04403	-1.41348	-2.92779

Path C catalyzed by phenylalanine

$^{2}RC_{ph}$

Fe	-3.37873	-0.25778	-0.07117
O	-1.81695	-0.18942	0.49944
S	-5.74747	-0.00626	-0.94689
H	-5.46676	-0.03156	-2.30022
N	-2.86148	-1.24067	-1.7533
C	-1.9941	-1.73396	-3.84678
C	-2.23469	-2.92441	-3.22238
H	-1.58979	-1.56247	-4.83326
H	-2.06889	-3.92404	-3.59611
N	-3.82672	-2.00663	0.81475
C	-3.62312	-3.27527	0.29912
C	-4.00298	-4.27314	1.27329
C	-4.43921	-3.60261	2.38113
H	-3.94214	-5.33976	1.11503
H	-4.80768	-4.0107	3.31073
N	-4.15191	0.73326	1.50818
C	-4.6065	0.17962	2.69766
C	-4.22614	2.1061	1.67615
C	-4.97849	1.22927	3.62042
C	-4.74057	2.41712	2.99186
H	-5.36837	1.06222	4.61362
H	-4.897	3.41767	3.36686

N	-3.2091	1.4936	-1.06577
C	-3.04909	3.76325	-1.53398
H	-3.11961	4.82989	-1.38073
C	-3.41083	2.77039	-0.54789
C	-2.38471	-0.68831	-2.92842
C	-2.76955	-2.61492	-1.9152
C	-4.32872	-2.1911	2.09008
C	-3.87088	3.05207	0.72568
H	-3.97059	4.09654	1.00017
C	-2.30662	0.67057	-3.20411
H	-1.89459	0.9612	-4.1635
C	-3.1323	-3.55845	-0.96765
H	-3.01824	-4.60349	-1.23541
C	-4.69078	-1.17546	2.96607
H	-5.07147	-1.46893	3.93854
C	-2.6122	3.09408	-2.64019
H	-2.24953	3.50331	-3.57128
C	-2.69833	1.68198	-2.34759
C	2.99127	-2.52916	1.7609
C	4.20998	-3.01249	2.23732
C	5.34858	-2.89629	1.41739
C	5.25106	-2.3023	0.12869
C	4.03716	-1.80453	-0.37385
C	2.94005	-1.93701	0.47495
H	6.7452	-3.81679	2.80971
H	4.29727	-3.46409	3.21953
C	6.652	-3.36529	1.82621
C	7.65593	-2.65943	-0.30218
C	7.73913	-3.2532	1.02217
H	8.71919	-3.60069	1.32168
O	6.36583	-2.19673	-0.68923
O	8.57564	-2.52475	-1.10745
O	4.01713	-1.17394	-1.6031
C	2.91909	-1.50872	-2.53723
H	1.99016	-1.02784	-2.23019
H	3.25464	-1.11735	-3.49697
H	2.8018	-2.5951	-2.59509
O	1.64914	-1.4967	0.20985
C	0.87731	-1.8163	1.35251
H	-0.15352	-1.5046	1.29108
C	1.64061	-2.43387	2.28937
H	1.30478	-2.77719	3.25523
C	5.4957	5.3803	0.22073
C	4.44132	4.69264	-0.38918
C	3.23403	4.47594	0.30098
C	3.11495	4.96731	1.61287

C	4.16852	5.65763	2.22339
C	5.36379	5.86748	1.52733
H	6.42198	5.53554	-0.32469
H	4.55082	4.2908	-1.38937
H	2.18826	4.80699	2.15817
H	4.05476	6.03003	3.23736
H	6.18317	6.40379	1.99655
C	2.07813	3.73042	-0.33533
C	2.35289	2.20762	-0.55508
H	1.83617	4.16043	-1.31744
H	1.1887	3.83503	0.29519
H	2.79378	1.81089	0.36503
C	1.02009	1.47981	-0.75725
O	0.64634	1.00621	-1.84518
O	0.28392	1.43348	0.37918
N	3.28272	1.98688	-1.65368
H	2.83997	2.07884	-2.56351
H	3.81018	1.12026	-1.58816
H	-0.54994	0.86889	0.32086

^{4}RC$_{ph}$

Fe	-3.37899	-0.25578	-0.07437
O	-1.8169	-0.22167	0.50224
S	-5.74506	-0.05281	-0.92772
H	-5.4752	-0.07507	-2.28325
N	-2.85795	-1.2429	-1.75495
C	-1.99465	-1.73846	-3.84954
C	-2.22773	-2.92822	-3.22114
H	-1.59405	-1.56769	-4.83765
H	-2.05811	-3.92806	-3.59259
N	-3.8117	-2.01334	0.82104
C	-3.60137	-3.28011	0.30681
C	-3.96415	-4.27942	1.28648
C	-4.39644	-3.6097	2.3966
H	-3.89486	-5.34589	1.13056
H	-4.75277	-4.01843	3.33064
N	-4.14641	0.72931	1.50931
C	-4.59284	0.17313	2.70185
C	-4.23536	2.10089	1.67441
C	-4.97185	1.22164	3.62318
C	-4.74823	2.41033	2.991
H	-5.35705	1.05314	4.61797
H	-4.91381	3.41016	3.36395
N	-3.22119	1.48547	-1.0679
C	-3.10064	3.75521	-1.55106
H	-3.1867	4.82151	-1.40353
C	-3.43999	2.76334	-0.5566

C	-2.38862	-0.69249	-2.93282
C	-2.76073	-2.61798	-1.91306
C	-4.30071	-2.198	2.09998
C	-3.89479	3.04677	0.71804
H	-4.00524	4.09143	0.98757
C	-2.325	0.66664	-3.21242
H	-1.92226	0.95951	-4.17509
C	-3.11614	-3.56146	-0.96319
H	-2.99785	-4.60622	-1.23039
C	-4.6654	-1.18139	2.9747
H	-5.03881	-1.4742	3.95021
C	-2.66195	3.08646	-2.6567
H	-2.31328	3.49597	-3.59302
C	-2.72425	1.67499	-2.35655
C	3.01209	-2.53543	1.75314
C	4.23454	-3.01105	2.22773
C	5.37244	-2.8817	1.40881
C	5.27052	-2.28255	0.12283
C	4.05278	-1.79229	-0.37776
C	2.9565	-1.93761	0.47001
H	6.77599	-3.79731	2.79738
H	4.32518	-3.46661	3.20779
C	6.67949	-3.34196	1.81598
C	7.67841	-2.61798	-0.30868
C	7.76596	-3.21721	1.01293
H	8.74873	-3.55806	1.31123
O	6.38466	-2.16418	-0.69419
O	8.59729	-2.47195	-1.1129
O	4.02768	-1.1561	-1.60411
C	2.93403	-1.49812	-2.5408
H	1.99949	-1.02966	-2.23156
H	3.26549	-1.09756	-3.49816
H	2.82925	-2.58543	-2.60483
O	1.66215	-1.50658	0.20648
C	0.89251	-1.83805	1.34727
H	-0.14077	-1.53448	1.28699
C	1.66049	-2.45378	2.28149
H	1.3271	-2.80453	3.24552
C	5.45329	5.41084	0.21928
C	4.40354	4.71391	-0.38809
C	3.20091	4.48412	0.30593
C	3.08164	4.97204	1.61909
C	4.13054	5.67162	2.2271
C	5.32122	5.8944	1.5272
H	6.37603	5.57614	-0.32914
H	4.51357	4.31514	-1.38945

H	2.1585	4.8017	2.16736
H	4.01673	6.04114	3.24211
H	6.13696	6.4379	1.99448
C	2.05014	3.72845	-0.32767
C	2.33808	2.20826	-0.5485
H	1.80176	4.1566	-1.30898
H	1.1614	3.82484	0.30516
H	2.78366	1.81484	0.37073
C	1.0113	1.46897	-0.74942
O	0.63842	0.99498	-1.83721
O	0.27878	1.41265	0.38935
H	-0.55061	0.84244	0.32986
N	3.26795	1.99604	-1.64846
H	2.82395	2.08617	-2.55781
H	3.8031	1.13403	-1.58453

^{2}TS-O$_{ph}$

Fe	2.93127	-0.53676	0.12035
O	1.42327	0.34803	-0.09408
S	4.77827	-2.10058	0.54398
H	4.51532	-3.01308	-0.45966
N	2.9585	-0.97777	-1.84934
C	2.47421	-1.88719	-3.92863
C	3.28186	-0.80823	-4.14192
H	2.04137	-2.55624	-4.65736
H	3.64667	-0.41853	-5.08085
N	4.17641	1.01193	-0.17958
C	4.668	1.446	-1.40205
C	5.55595	2.57073	-1.2074
C	5.61619	2.80658	0.13776
H	6.06713	3.09394	-2.00218
H	6.1856	3.56098	0.66059
N	3.04014	-0.21015	2.10493
C	3.75128	0.78384	2.76466
C	2.37199	-0.92016	3.09118
C	3.51769	0.69541	4.18953
C	2.66341	-0.35179	4.38993
H	3.96041	1.34941	4.9265
H	2.26903	-0.72441	5.32395
N	1.80848	-2.19841	0.43861
C	0.49734	-3.81266	1.47004
H	0.00266	-4.3499	2.26575
C	1.31584	-2.6331	1.66074
C	2.27239	-1.98809	-2.49861
C	3.57881	-0.23936	-2.8432
C	4.76238	1.83076	0.77637
C	1.56455	-2.03029	2.88281

H	1.10476	-2.47752	3.75785
C	1.50653	-2.96738	-1.87766
H	1.02634	-3.70166	-2.51516
C	4.37597	0.87588	-2.63472
H	4.81918	1.33869	-3.51041
C	4.55815	1.72724	2.14631
H	5.07183	2.44017	2.78278
C	0.48602	-4.08343	0.13263
H	-0.02022	-4.8852	-0.38444
C	1.29411	-3.07015	-0.51309
C	-0.80323	2.61673	1.17252
C	-1.90165	2.69682	2.03417
C	-3.17866	2.85281	1.47084
C	-3.33469	2.92052	0.0605
C	-2.24534	2.85533	-0.83809
C	-0.99844	2.72071	-0.23428
H	-4.27615	2.88527	3.34967
H	-1.78505	2.62316	3.10929
C	-4.37997	2.93479	2.26989
C	-5.79131	3.1278	0.26936
C	-5.60805	3.06761	1.70939
H	-6.51125	3.13002	2.30193
O	-4.5931	3.03077	-0.50647
O	-6.85117	3.24741	-0.33904
O	-2.53981	2.92949	-2.17303
C	-1.49099	2.72057	-3.1883
H	-1.07108	1.71771	-3.09661
H	-2.02189	2.83522	-4.13224
H	-0.7115	3.4789	-3.09306
O	0.23467	2.62064	-0.88569
C	1.19565	2.35244	0.11024
H	2.21605	2.47703	-0.20169
C	0.60351	2.42764	1.36789
H	1.11864	2.29782	2.30576
C	-7.13785	-2.99498	-0.2166
C	-5.92999	-2.63097	-0.82079
C	-4.70799	-2.82036	-0.14803
C	-4.73249	-3.38441	1.13959
C	-5.94049	-3.75225	1.74361
C	-7.14888	-3.55882	1.06559
H	-8.07201	-2.83794	-0.7477
H	-5.9199	-2.17062	-1.80154
H	-3.79589	-3.53721	1.6702
H	-5.93686	-4.18888	2.73822
H	-8.08821	-3.84402	1.52976
C	-3.38668	-2.43021	-0.77941

C	-3.20636	-0.89038	-0.97152
H	-3.27902	-2.89261	-1.77036
H	-2.56453	-2.801	-0.156
H	-3.51165	-0.39585	-0.04529
C	-1.72001	-0.59243	-1.18345
O	-1.21389	-0.43729	-2.31124
O	-1.02529	-0.53687	-0.0272
H	-0.04484	-0.25008	-0.1164
N	-4.026	-0.39048	-2.07149
H	-3.59887	-0.56881	-2.97675
H	-4.29413	0.5851	-1.98196

^{4}TS-O$_{ph}$

Fe	-2.86879	0.55817	0.11275
O	-1.42255	-0.42622	-0.13445
S	-4.81704	2.02083	0.62943
H	-4.3253	3.21292	0.13142
N	-2.6414	1.3658	-1.71382
C	-1.93984	2.6793	-3.49394
C	-2.66548	1.65118	-4.01891
H	-1.44679	3.48725	-4.01362
H	-2.89148	1.45079	-5.05582
N	-4.11929	-0.84873	-0.63503
C	-4.40445	-1.05218	-1.97152
C	-5.31872	-2.16211	-2.12025
C	-5.59671	-2.62197	-0.86175
H	-5.69698	-2.52576	-3.06433
H	-6.24385	-3.43765	-0.5744
N	-3.23563	-0.16411	1.9738
C	-4.06301	-1.22443	2.32829
C	-2.66349	0.28166	3.15097
C	-3.99514	-1.44586	3.75716
C	-3.12415	-0.5231	4.2637
H	-4.54926	-2.20601	4.2882
H	-2.82844	-0.37552	5.29209
N	-1.82997	2.0776	0.89569
C	-0.65088	3.41654	2.38443
H	-0.24132	3.75953	3.32311
C	-1.46157	2.22872	2.23115
C	-1.92645	2.49431	-2.05701
C	-3.10797	0.83309	-2.90686
C	-4.84455	-1.80195	0.06044
C	-1.81958	1.38025	3.26392
H	-1.44448	1.61896	4.25356
C	-1.28641	3.33541	-1.15287
H	-0.76205	4.19546	-1.55509
C	-3.91728	-0.28321	-3.02544

H	-4.22085	-0.57071	-4.02677
C	-4.82258	-1.9711	1.44194
H	-5.43545	-2.76519	1.85567
C	-0.5215	3.9826	1.1493
H	0.01645	4.87862	0.87667
C	-1.23924	3.14261	0.21675
C	0.78802	-2.67653	1.16439
C	1.89021	-2.76719	2.01981
C	3.16676	-2.90963	1.44898
C	3.31705	-2.9559	0.03838
C	2.2224	-2.88417	-0.85473
C	0.97813	-2.76069	-0.24455
H	4.27168	-2.96675	3.32264
H	1.77816	-2.71052	3.09651
C	4.37127	-2.99866	2.24173
C	5.77563	-3.1529	0.23306
C	5.59799	-3.11702	1.67437
H	6.50377	-3.1848	2.26239
O	4.57422	-3.05067	-0.53593
O	6.83431	-3.25561	-0.38135
O	2.51262	-2.9431	-2.19164
C	1.45693	-2.74264	-3.20058
H	1.02775	-1.74433	-3.1042
H	1.984	-2.84953	-4.14767
H	0.68553	-3.50911	-3.10332
O	-0.25904	-2.65555	-0.89081
C	-1.21646	-2.38359	0.11082
H	-2.23439	-2.54056	-0.19537
C	-0.61834	-2.49603	1.36936
H	-1.12919	-2.38857	2.31248
C	7.05186	3.10893	-0.20804
C	5.85467	2.71055	-0.81169
C	4.62746	2.87152	-0.14112
C	4.6355	3.44156	1.14404
C	5.83283	3.84374	1.74748
C	7.04658	3.67903	1.07144
H	7.99045	2.97368	-0.7373
H	5.85739	2.24453	-1.78977
H	3.69458	3.57105	1.67328
H	5.81678	4.28433	2.74021
H	7.97761	3.99083	1.53522
C	3.31758	2.44558	-0.77305
C	3.17524	0.9016	-0.96281
H	3.1981	2.90392	-1.76457
H	2.486	2.79503	-0.14955
H	3.4833	0.41613	-0.03273

C	1.69747	0.57005	-1.18854
O	1.20841	0.39784	-2.32097
O	0.99136	0.5072	-0.03977
H	0.01898	0.18833	-0.13924
N	4.01439	0.41725	-2.05488
H	3.58735	0.57979	-2.96332
H	4.303	-0.55197	-1.95914

$^{2}INT_{ph}$

Fe	-3.12594	0.26916	0.55617
O	-1.45123	-0.45575	-0.60075
S	-4.83946	1.08081	1.83745
H	-4.6526	2.4251	1.57591
N	-3.54368	1.49639	-0.98497
C	-3.65783	3.33865	-2.3956
C	-4.54376	2.41374	-2.8695
H	-3.43694	4.31908	-2.79202
H	-5.19121	2.48671	-3.73133
N	-4.31359	-1.16315	-0.23563
C	-5.15101	-1.01391	-1.33415
C	-5.94133	-2.21332	-1.51744
C	-5.59096	-3.08271	-0.52411
H	-6.66928	-2.35315	-2.30349
H	-5.97624	-4.0742	-0.33506
N	-2.5897	-1.03	2.00014
C	-3.07245	-2.31271	2.21654
C	-1.73055	-0.76178	3.05588
C	-2.48884	-2.86419	3.42177
C	-1.66375	-1.90773	3.93985
H	-2.70168	-3.84918	3.81139
H	-1.06337	-1.95508	4.83683
N	-1.82822	1.63798	1.25718
C	-0.2956	2.72987	2.61184
H	0.3574	2.90638	3.45502
C	-1.06917	1.52181	2.40929
C	-3.03839	2.77015	-1.21778
C	-4.47445	1.26582	-1.98914
C	-4.58456	-2.42502	0.28139
C	-1.02686	0.41627	3.24682
H	-0.38354	0.47672	4.11817
C	-2.10152	3.41578	-0.42728
H	-1.79352	4.41266	-0.72396
C	-5.22073	0.10604	-2.15006
H	-5.91818	0.0744	-2.9804
C	-3.99527	-2.96883	1.41336
H	-4.29819	-3.96796	1.70782
C	-0.58823	3.57315	1.57125

H	-0.22518	4.57664	1.39841
C	-1.54369	2.88598	0.72755
C	0.31701	-2.14262	-1.07922
C	1.20153	-2.9404	-0.37723
C	2.57479	-2.85273	-0.69606
C	3.0077	-1.94923	-1.69382
C	2.11588	-1.14118	-2.43715
C	0.77501	-1.28418	-2.10548
H	3.26272	-4.34189	0.7323
H	0.86687	-3.60182	0.41383
C	3.58425	-3.63211	-0.02402
C	5.37187	-2.53018	-1.29099
C	4.90366	-3.49118	-0.31041
H	5.6694	-4.06799	0.19092
O	4.35215	-1.80576	-1.9784
O	6.5409	-2.27553	-1.58003
O	2.6569	-0.34065	-3.40771
C	1.90612	0.82087	-3.90102
H	1.60205	1.45057	-3.063
H	2.61603	1.33859	-4.54576
H	1.03125	0.50625	-4.47193
O	-0.29901	-0.60984	-2.73644
C	-1.47329	-0.93517	-1.99834
H	-2.38493	-0.79116	-2.55461
C	-1.1376	-1.94385	-0.96458
H	-1.83494	-2.64614	-0.53429
C	6.64552	-0.74987	1.57455
C	6.4114	0.35311	0.74665
C	5.67166	1.45627	1.20974
C	5.16282	1.42177	2.51935
C	5.39492	0.31888	3.34969
C	6.14004	-0.76884	2.8806
H	7.22406	-1.58517	1.19365
H	6.80891	0.35145	-0.2643
H	4.58478	2.26532	2.88492
H	5.0005	0.31277	4.36163
H	6.32831	-1.62086	3.52699
C	5.43901	2.65662	0.31233
C	4.35057	2.45008	-0.76437
H	6.35484	2.91108	-0.2323
H	5.16508	3.52784	0.91825
H	4.60146	1.58285	-1.38225
C	2.97142	2.1586	-0.18009
O	2.18767	1.29698	-0.59938
O	2.61164	3.04369	0.81135
H	1.68539	2.87955	1.11585

N	4.28814	3.62804	-1.63571
H	4.01984	4.48893	-1.17416
H	3.84179	3.49049	-2.53292

[4]INT$_{ph}$

Fe	-3.28314	0.33926	0.53434
O	-1.29835	-0.68176	-0.70695
S	-5.14009	1.34947	1.82393
H	-5.8161	1.91755	0.76013
N	-3.6643	1.30923	-1.18834
C	-3.79472	2.96222	-2.81102
C	-4.60113	1.93482	-3.21672
H	-3.60384	3.90018	-3.31176
H	-5.20036	1.86867	-4.11317
N	-4.35772	-1.24211	-0.13995
C	-5.12307	-1.29954	-1.2985
C	-5.85808	-2.54488	-1.34124
C	-5.54854	-3.23438	-0.20324
H	-6.52711	-2.83461	-2.13846
H	-5.91373	-4.20021	0.1143
N	-2.68427	-0.75805	2.10955
C	-3.17185	-2.00167	2.49216
C	-1.79658	-0.37091	3.10616
C	-2.57965	-2.39443	3.74994
C	-1.73095	-1.39091	4.12683
H	-2.79497	-3.31844	4.26654
H	-1.11451	-1.33257	5.01198
N	-1.93055	1.76238	1.02237
C	-0.39404	3.03828	2.20069
H	0.2767	3.3244	2.99845
C	-1.13715	1.79672	2.15727
C	-3.20642	2.57204	-1.55154
C	-4.51983	0.90462	-2.20802
C	-4.61688	-2.42173	0.54807
C	-1.07291	0.81064	3.12866
H	-0.41132	0.97728	3.9715
C	-2.3023	3.34001	-0.83515
H	-2.03519	4.30919	-1.24182
C	-5.19521	-0.30694	-2.26356
H	-5.83702	-0.4837	-3.11982
C	-4.07047	-2.77526	1.77129
H	-4.36735	-3.72701	2.19822
C	-0.74679	3.75561	1.08765
H	-0.42151	4.74428	0.79609
C	-1.70481	2.95498	0.35454
C	0.54112	-2.35103	-0.92512
C	1.4069	-3.04981	-0.10478

C	2.79318	-2.95273	-0.35932
C	3.25631	-2.13686	-1.4167
C	2.38597	-1.43167	-2.27957
C	1.03162	-1.58201	-2.00681
H	3.44015	-4.27849	1.2386
H	1.04766	-3.64297	0.72875
C	3.784	-3.63466	0.43448
C	5.60909	-2.6065	-0.84093
C	5.11329	-3.48395	0.2023
H	5.86517	-3.98871	0.79424
O	4.6109	-1.98275	-1.64645
O	6.78578	-2.34377	-1.09108
O	2.96269	-0.71177	-3.29325
C	2.19526	0.33317	-3.981
H	1.80453	1.0476	-3.25454
H	2.92369	0.80141	-4.64288
H	1.37744	-0.09971	-4.55909
O	-0.01986	-1.00733	-2.75627
C	-1.22947	-1.28312	-2.03972
H	-2.10692	-1.22876	-2.6638
C	-0.92275	-2.17627	-0.896
H	-1.62032	-2.86024	-0.43585
C	6.55842	-0.40684	1.82306
C	6.35292	0.58041	0.85365
C	5.57219	1.71566	1.13692
C	4.99492	1.83248	2.41303
C	5.19817	0.84543	3.38474
C	5.98289	-0.27592	3.0932
H	7.17013	-1.26936	1.57961
H	6.80557	0.46317	-0.12689
H	4.38748	2.70322	2.64187
H	4.75088	0.95614	4.3683
H	6.14856	-1.03771	3.84913
C	5.3694	2.78939	0.08521
C	4.32892	2.43385	-1.00098
H	6.30565	2.98386	-0.4491
H	5.0633	3.72745	0.56233
H	4.61498	1.50091	-1.49535
C	2.93051	2.19417	-0.43955
O	2.17861	1.26643	-0.76406
O	2.51414	3.20122	0.40453
H	1.58095	3.05595	0.69472
N	4.29014	3.49352	-2.01377
H	3.98908	4.39951	-1.67526
H	3.8879	3.23764	-2.90587

$^{2}P_{ph}$

Fe	-3.04822	0.0168	0.31965
O	-0.71228	-0.44721	-0.346
S	-5.17953	0.53225	0.99356
H	-5.86123	-0.07778	-0.04323
N	-2.50285	-0.60323	2.15251
C	-2.27245	-1.89606	4.06983
C	-1.80337	-0.64854	4.36807
H	-2.328	-2.7625	4.71293
H	-1.40036	-0.2902	5.30414
N	-2.41702	1.86197	0.78237
C	-1.9326	2.31322	2.00162
C	-1.75375	3.7485	1.9637
C	-2.13772	4.16601	0.72086
H	-1.39407	4.344	2.79012
H	-2.16351	5.17363	0.33079
N	-3.37951	0.57219	-1.57932
C	-3.31586	1.87043	-2.07063
C	-3.82546	-0.21212	-2.63992
C	-3.70124	1.88984	-3.465
C	-4.00887	0.60691	-3.81757
H	-3.72809	2.77742	-4.08015
H	-4.34043	0.23665	-4.77669
N	-3.54271	-1.87322	-0.18578
C	-4.39056	-3.72459	-1.30894
H	-4.78049	-4.31757	-2.12347
C	-4.00913	-2.33498	-1.41497
C	-2.7156	-1.86649	2.69223
C	-1.96305	0.16101	3.17998
C	-2.54142	2.98705	-0.01826
C	-4.11123	-1.56806	-2.56712
H	-4.47618	-2.05439	-3.46535
C	-3.2582	-2.9513	2.01849
H	-3.3814	-3.87395	2.57575
C	-1.68777	1.51741	3.10875
H	-1.29028	1.99759	3.99622
C	-2.94897	2.99153	-1.34281
H	-2.97484	3.94759	-1.85478
C	-4.17323	-4.10385	-0.01387
H	-4.34849	-5.06936	0.43815
C	-3.64616	-2.95404	0.6865
C	1.2174	-2.06556	-0.03973
C	2.07135	-2.51909	0.9498
C	3.4515	-2.57955	0.65843
C	3.92304	-2.16077	-0.60771
C	3.06421	-1.69731	-1.63855
C	1.71256	-1.68322	-1.30902

H	4.07709	-3.39504	2.57653
H	1.70432	-2.82116	1.92416
C	4.42935	-3.05384	1.60746
C	6.2619	-2.61998	0.03306
C	5.75442	-3.0801	1.31455
H	6.49705	-3.43319	2.01802
O	5.26894	-2.19785	-0.90368
O	7.43757	-2.56121	-0.31846
O	3.64398	-1.37844	-2.82973
C	2.91166	-0.58132	-3.83449
H	2.50714	0.32187	-3.3769
H	3.6723	-0.34383	-4.57655
H	2.11296	-1.17755	-4.27999
O	0.65589	-1.35469	-2.19211
C	-0.55042	-1.44723	-1.44915
H	-1.41952	-1.60781	-2.0634
C	-0.2471	-1.93103	-0.08363
H	-0.93346	-2.46528	0.55334
C	6.42426	2.28526	0.14877
C	5.31928	3.14094	0.12435
C	4.14599	2.8277	0.83492
C	4.11368	1.6385	1.58104
C	5.22338	0.78557	1.61712
C	6.38032	1.10166	0.89634
H	7.31807	2.53925	-0.41239
H	5.36558	4.06298	-0.44978
H	3.21217	1.38295	2.13039
H	5.18462	-0.12472	2.20698
H	7.23476	0.43255	0.90862
C	2.94523	3.75177	0.78232
C	2.17713	3.70444	-0.55802
H	3.25273	4.79502	0.9178
H	2.24617	3.50676	1.58959
H	2.86688	3.91985	-1.37898
C	1.58469	2.33006	-0.86154
O	1.69549	1.74777	-1.95067
O	0.82531	1.8449	0.16384
H	0.32113	1.01608	-0.06927
N	1.12805	4.73277	-0.56404
H	0.39692	4.57413	0.12271
H	0.75254	4.94119	-1.48261

$^4P_{ph}$

Fe	-3.32324	0.0089	0.33467
O	-0.3867	-0.49139	-0.435
S	-5.64754	0.49233	0.99274
H	-6.25507	-0.38566	0.11431

N	-2.65928	-0.45458	2.18311
C	-2.32979	-1.5734	4.19229
C	-1.96645	-0.27143	4.39201
H	-2.31585	-2.38907	4.90043
H	-1.59694	0.18974	5.29622
N	-2.69611	1.89883	0.60643
C	-2.226	2.46681	1.78326
C	-2.04882	3.88969	1.60812
C	-2.4147	4.18541	0.32423
H	-1.69753	4.56273	2.37643
H	-2.42881	5.14982	-0.16283
N	-3.55925	0.3822	-1.63253
C	-3.53276	1.6314	-2.24439
C	-3.94063	-0.51585	-2.62465
C	-3.89263	1.50614	-3.63789
C	-4.13809	0.18315	-3.87388
H	-3.94419	2.3309	-4.33336
H	-4.43272	-0.28857	-4.7999
N	-3.58364	-1.9572	-0.03836
C	-4.23457	-3.94973	-1.03964
H	-4.55791	-4.62976	-1.81425
C	-3.98775	-2.54205	-1.23758
C	-2.7638	-1.68773	2.81881
C	-2.18114	0.42718	3.14566
C	-2.80892	2.94375	-0.30164
C	-4.13837	-1.87605	-2.44476
H	-4.45289	-2.45747	-3.30444
C	-3.20473	-2.86234	2.22976
H	-3.24911	-3.75075	2.85012
C	-1.97526	1.78438	2.96209
H	-1.60598	2.35643	3.80584
C	-3.20422	2.82433	-1.62368
H	-3.24273	3.72858	-2.2209
C	-3.98978	-4.2225	0.27831
H	-4.07204	-5.16961	0.79128
C	-3.58146	-2.98721	0.90175
C	1.46614	-2.11944	0.06414
C	2.31865	-2.46019	1.09917
C	3.69931	-2.56383	0.81891
C	4.17477	-2.29755	-0.48672
C	3.31884	-1.94305	-1.56035
C	1.96661	-1.88947	-1.23917
H	4.32332	-3.12608	2.8269
H	1.95175	-2.63905	2.10372
C	4.67587	-2.91604	1.8213
C	6.51003	-2.71222	0.20108

C	6.00073	-2.98807	1.53391
H	6.74138	-3.2521	2.27734
O	5.52067	-2.37833	-0.77583
O	7.68234	-2.73938	-0.16322
O	3.88948	-1.74858	-2.78406
C	3.21468	-0.89504	-3.78503
H	2.89209	0.04118	-3.32659
H	3.98264	-0.72274	-4.5375
H	2.36217	-1.42225	-4.21676
O	0.92061	-1.65005	-2.1599
C	-0.28837	-1.60055	-1.40648
H	-1.16597	-1.80168	-1.99851
C	0.00503	-1.93448	0.00959
H	-0.69905	-2.36263	0.70638
C	6.50534	2.90619	0.42609
C	5.33486	3.65647	0.27682
C	4.10412	3.18051	0.76315
C	4.07728	1.93333	1.41056
C	5.24798	1.18161	1.56411
C	6.46524	1.66363	1.07032
H	7.44588	3.28899	0.04209
H	5.37356	4.62322	-0.21892
H	3.13259	1.55268	1.78805
H	5.21072	0.22055	2.06791
H	7.37185	1.07768	1.18294
C	2.83658	3.98873	0.5661
C	2.16944	3.77846	-0.8153
H	3.04479	5.06129	0.64338
H	2.10256	3.73792	1.34034
H	2.89108	4.00875	-1.60403
C	1.74135	2.33064	-1.0391
O	2.05661	1.64212	-2.02027
O	0.88932	1.89393	-0.06564
H	0.50235	0.99078	-0.24797
N	1.02648	4.69057	-0.95576
H	0.26217	4.48415	-0.31933
H	0.70729	4.81658	-1.90993

Reactive metabolites formation reactions

8-MP epoxide

O	0.30885	-1.30181	1.57381
C	2.63025	-3.02509	1.11868
C	3.87255	-3.11436	0.50393
C	4.36591	-4.39331	0.20092
C	3.61997	-5.55226	0.51263
C	2.35644	-5.48584	1.13671

C	1.90224	-4.20219	1.42071
H	6.23461	-3.72355	-0.68576
H	4.45339	-2.23398	0.26013
C	5.64501	-4.60001	-0.43748
C	5.35539	-7.04235	-0.41072
C	6.10785	-5.83492	-0.72339
H	7.06399	-5.99497	-1.20087
O	4.09267	-6.81405	0.22062
O	5.68053	-8.19765	-0.6252
O	1.72758	-6.67527	1.38947
C	0.40761	-6.66838	2.04003
H	-0.32894	-6.13447	1.43501
H	0.14642	-7.72301	2.11853
H	0.45716	-6.21041	3.03072
O	0.68839	-3.86212	2.03346
C	0.67896	-2.448	2.10502
H	-0.19972	-2.0254	2.55693
C	1.80491	-1.91724	1.57661
H	2.04814	-0.87067	1.50639

TS_{OMe}

O	-0.00598	-1.65997	-0.40174
C	-1.913	-0.12648	-0.65707
C	-1.60971	1.15006	-0.48743
O	-0.60735	1.27449	0.60119
C	-0.35988	0.06084	1.06111
H	1.37489	-0.99642	-0.01872
C	-1.07208	-0.99762	0.27592
H	-1.68499	-1.67159	0.88962
O	1.98763	-0.29196	0.39312
C	2.52373	0.59199	-0.61763
H	3.18198	1.3033	-0.12371
H	3.10077	0.03258	-1.35936
H	1.73277	1.14298	-1.13879
H	0.31831	-0.02428	1.88772
C	-4.7142	1.33116	-4.38316
C	-4.17013	0.30705	-3.64847
C	-3.20228	0.57561	-2.64187
C	-2.80602	1.91748	-2.40619
C	-4.31836	2.6702	-4.14773
H	-2.93643	-1.50273	-2.05652
H	-5.46297	1.12599	-5.16241
H	-4.47161	-0.73644	-3.82445
C	-2.62219	-0.46556	-1.86622
C	-1.83816	2.18519	-1.39927
O	-1.44607	3.54117	-1.17017
C	-2.33997	4.60873	-1.49599

H	-2.22302	4.86972	-2.52707
H	-3.34798	4.29638	-1.31925
H	-2.11776	5.45889	-0.88546
O	-3.38582	2.95853	-3.18166
O	-4.90411	3.71411	-4.93008

P_{OMe}

C	-0.93015	-0.96412	0.42259
C	0.1169	-1.81924	0.17325
C	1.41136	-1.28058	0.03434
C	1.60415	0.11254	0.15223
C	0.53754	0.99566	0.40964
C	-0.71745	0.41082	0.53189
H	2.45446	-3.1518	-0.33196
H	-0.04109	-2.88699	0.07499
C	2.57587	-2.07731	-0.23546
C	3.99008	-0.07781	-0.25241
C	3.79601	-1.51504	-0.369
H	4.68757	-2.09259	-0.57141
O	2.83357	0.67133	0.01207
O	5.0259	0.51868	-0.36157
O	0.77105	2.32591	0.56195
C	0.16518	3.17991	-0.42253
H	-0.92222	3.07886	-0.41686
H	0.44543	4.19499	-0.14597
H	0.55677	2.95222	-1.41874
O	-1.8697	1.11033	0.77339
C	-2.97322	0.23527	0.49589
H	-3.76793	0.48373	1.20814
C	-2.39335	-1.21033	0.68016
H	-2.53496	-1.51	1.72759
O	-3.37423	0.49423	-0.81146
O	-2.96159	-2.17372	-0.18993
H	-3.57284	-2.71703	0.31448
C	-4.74899	0.25374	-1.08577
H	-5.3921	0.77718	-0.36508
H	-4.93729	0.65118	-2.08232
H	-4.97925	-0.81486	-1.07721

TS_{SMe}

O	-0.00598	-1.65997	-0.40174
C	-1.913	-0.12648	-0.65707
C	-1.60971	1.15006	-0.48743
O	-0.60735	1.27449	0.60119
C	-0.35988	0.06084	1.06111
H	1.48449	-1.08265	-0.66367
C	-1.07208	-0.99762	0.27592
H	-1.68499	-1.67159	0.88962

C	2.74554	1.22141	-0.99078
H	3.40379	1.93272	-0.49686
H	3.32258	0.66199	-1.73251
H	1.95459	1.77239	-1.51194
H	0.31831	-0.02428	1.88772
C	-4.7142	1.33116	-4.38316
C	-4.17013	0.30705	-3.64847
C	-3.20228	0.57561	-2.64187
C	-2.80602	1.91748	-2.40619
C	-4.31836	2.6702	-4.14773
H	-2.93643	-1.50273	-2.05652
H	-5.46297	1.12599	-5.16241
H	-4.47161	-0.73644	-3.82445
C	-2.62219	-0.46556	-1.86622
C	-1.83816	2.18519	-1.39927
O	-1.44607	3.54117	-1.17017
C	-2.33997	4.60873	-1.49599
H	-2.22302	4.86972	-2.52707
H	-3.34798	4.29638	-1.31925
H	-2.11776	5.45889	-0.88546
O	-3.38582	2.95853	-3.18166
O	-4.90411	3.71411	-4.93008
S	2.08553	0.13314	0.25359

PS$_{Me}$

C	2.63025	-3.02509	1.11868
C	3.87255	-3.11436	0.50393
C	4.36591	-4.39331	0.20092
C	3.61997	-5.55226	0.51263
C	2.35644	-5.48584	1.13671
C	1.90224	-4.20219	1.42071
H	6.23461	-3.72355	-0.68576
H	4.45339	-2.23398	0.26013
C	5.64501	-4.60001	-0.43748
C	5.35539	-7.04235	-0.41072
C	6.10785	-5.83492	-0.72339
H	7.06399	-5.99497	-1.20087
O	4.09267	-6.81405	0.22062
O	5.68053	-8.19765	-0.6252
O	1.72758	-6.67527	1.38947
C	0.40761	-6.66838	2.04003
H	-0.32894	-6.13447	1.43501
H	0.14642	-7.72301	2.11853
H	0.45716	-6.21041	3.03072
O	0.68839	-3.86212	2.03346
C	0.67896	-2.448	2.10502
H	-0.16814	-2.07661	1.56708

C	1.80491	-1.91724	1.57661
H	1.5563	-1.28278	0.75166
O	2.50471	-1.16833	2.57376
H	2.46478	-0.23347	2.35921
C	-0.49331	-1.10917	3.61774
H	-1.43112	-1.61849	3.54021
H	-0.42362	-0.63372	4.57376
H	-0.42352	-0.37096	2.84633
S	0.5736	-2.05051	3.47462

Phenylalanine

C	2.88041	-0.83264	0.76367
C	1.62387	-1.28122	0.367
C	0.81741	-0.50911	-0.47731
C	1.30674	0.72463	-0.918
C	2.5646	1.17692	-0.52395
C	3.35495	0.40012	0.31904
H	3.49144	-1.44707	1.41565
H	1.26583	-2.24691	0.71067
H	0.69548	1.33386	-1.57515
H	2.92796	2.13516	-0.87875
H	4.33462	0.74988	0.62455
C	-0.55458	-0.99856	-0.88698
C	-1.64849	-0.76665	0.17361
H	-0.53633	-2.07508	-1.07557
H	-0.86224	-0.51257	-1.81697
H	-1.35561	-1.24866	1.10836
C	-1.84364	0.70788	0.5206
O	-1.88061	1.17062	1.63084
O	-2.06238	1.46601	-0.58993
H	-2.22713	2.36796	-0.27701
N	-2.90337	-1.38137	-0.27928
H	-3.26579	-0.87793	-1.08403
H	-3.60831	-1.33032	0.44989

TS_{Phe}

O	-3.20591	-1.76968	0.42703
C	-1.00653	-1.21276	-0.51466
C	-0.81684	0.14672	-0.69716
O	-2.04092	0.7822	-1.04597
C	-2.9559	-0.13365	-1.12516
H	-3.9384	-0.45491	0.89794
C	-2.50159	-1.51485	-0.70365
H	-2.64643	-2.24746	-1.52623
O	-4.24965	0.50328	0.87879
C	-3.50698	1.24977	1.82214
H	-2.55342	1.60625	1.40103
H	-3.93934	0.19621	-1.42249

C	3.69823	-1.4676	0.55095
C	2.505	-2.08277	0.42277
C	1.3186	-1.35926	0.04003
C	1.46695	0.02431	-0.18468
C	3.85268	-0.04159	0.30737
H	-0.04137	-3.0452	0.06271
H	4.59863	-1.99334	0.83806
H	2.41652	-3.14833	0.60774
C	0.06509	-1.98349	-0.12537
C	0.37259	0.84173	-0.55226
O	0.40638	2.16156	-0.82677
C	1.16127	3.05159	0.02326
H	0.94795	2.84687	1.07561
H	2.22945	2.96445	-0.16398
H	0.80847	4.04851	-0.23359
O	2.67856	0.63339	-0.06864
O	4.85793	0.60574	0.39141
O	-4.26788	2.39709	2.20889
C	-3.18792	0.42022	3.07978
H	-2.57038	-0.41134	2.81136
N	-2.47984	1.2617	4.0552
H	-2.87863	1.12795	4.96243
H	-1.51298	1.00718	4.07525
C	-4.49945	-0.09404	3.70189
H	-5.00416	-0.72428	2.99978
H	-5.12626	0.73684	3.95019
C	-4.18244	-0.89706	4.97714
C	-4.55916	-0.40096	6.22551
C	-3.51881	-2.12037	4.88416
C	-4.27167	-1.12775	7.38065
H	-5.08165	0.56388	6.29853
C	-3.23204	-2.84798	6.03948
H	-3.22199	-2.51161	3.90031
C	-3.60822	-2.35184	7.28762
H	-4.56802	-0.73649	8.36471
H	-2.70915	-3.81268	5.96583
H	-3.3817	-2.92474	8.19854

P_{Phe}

O	0.49069	-3.49128	0.74929
C	2.2128	-2.02484	-0.33849
C	1.735	-0.93142	-1.05544
O	0.46707	-1.27018	-1.66139
C	0.17202	-2.50549	-1.32597
C	1.19174	-3.15817	-0.43587
H	1.62639	-4.05142	-0.91088
O	-0.70564	-2.14357	-0.21532

C	-0.52296	-0.92731	0.5006
H	-0.77116	-2.89492	-1.66568
C	5.89538	0.81004	1.04375
C	5.27739	-0.3946	1.07185
C	4.04799	-0.63945	0.35478
C	3.51054	0.43505	-0.38644
C	5.36217	1.93225	0.29801
H	3.77294	-2.70077	0.97548
H	6.81315	0.99897	1.57806
H	5.70314	-1.2083	1.64488
C	3.38252	-1.88889	0.37812
C	2.31072	0.31947	-1.12156
O	1.76091	1.31084	-1.89255
C	1.38306	2.57997	-1.22469
H	0.7519	2.35391	-0.36728
H	2.27458	3.12721	-0.93323
H	0.82657	3.12314	-1.98036
O	4.14414	1.66756	-0.41538
O	5.82647	3.0619	0.21428
O	0.34717	-0.03644	-0.00765
C	-2.03519	-0.20091	0.54938
H	-2.65013	-0.9993	0.96129
N	-2.06083	0.92837	1.4056
H	-1.4753	1.70799	1.1497
H	-2.15375	0.7791	2.39672
C	-2.46021	0.11941	-0.89842
H	-2.11917	-0.69866	-1.52965
H	-1.93495	1.02132	-1.22056
C	-3.95823	0.29044	-1.0353
C	-4.5807	1.5248	-0.79265
C	-4.76241	-0.80342	-1.39429
C	-5.96541	1.66337	-0.90902
H	-3.97646	2.3748	-0.5048
C	-6.14825	-0.66925	-1.50914
H	-4.29566	-1.76233	-1.58471
C	-6.75437	0.56624	-1.26742
H	-6.42771	2.62397	-0.72166
H	-6.75071	-1.52352	-1.78976
H	-7.82718	0.67386	-1.35876
H	1.11885	-3.68771	1.44817

TS_{ro}

O	1.26977	0.22506	0.68276
C	-1.25722	1.28429	0.09893
C	-1.80255	-0.13965	0.2098
O	-0.77285	-1.0305	-0.11648
C	0.60823	-0.07972	-0.3936

H	1.05228	-0.63968	-1.22029
C	0.06065	1.28362	-0.37065
H	0.66917	2.13311	-0.63573
C	-6.61308	1.60908	0.33206
C	-4.29954	0.87863	0.38521
C	-3.87691	2.20232	0.09817
C	-4.85377	3.22166	-0.06908
C	-6.19088	2.92996	0.04547
C	-3.1878	-0.14074	0.52204
C	-2.27498	2.27003	0.00476
H	-4.51571	4.24472	-0.29162
H	-6.9493	3.71616	-0.08334
H	-2.16642	3.50321	-0.23755
O	-5.6906	0.60611	0.49838
O	-3.62711	-1.47047	0.81141
C	-3.59319	-1.68752	2.22443
H	-4.56045	-1.99741	2.561
H	-2.87592	-2.44841	2.45129
H	-3.31782	-0.77913	2.71831
O	-8.01266	1.33831	0.44512
**P_{ro}**			
C	-1.4582	-1.17051	-0.1203
C	-0.32187	-1.94245	-0.02286
C	0.93045	-1.30026	-0.01394
C	0.99778	0.10615	-0.08615
C	-0.15548	0.91284	-0.19503
C	-1.36643	0.23071	-0.23203
H	2.15389	-3.09086	0.14777
H	-0.37501	-3.02205	0.05546
C	2.17871	-2.00734	0.0871
C	3.42213	0.10163	0.03194
C	3.35702	-1.3508	0.10616
H	4.30891	-1.85867	0.17958
O	2.18627	0.75971	-0.06298
O	4.4127	0.77768	0.04674
O	0.00013	2.25113	-0.32841
C	-0.82726	3.10003	0.48256
H	-0.73615	2.82818	1.53847
H	-0.44294	4.10714	0.33193
H	-1.87315	3.04868	0.177
O	-2.58801	0.83056	-0.41807
C	-2.90097	-1.49414	-0.14679
H	-3.49276	-1.71014	0.73348
C	-3.12978	-2.09702	-1.54528
O	-4.233	-2.48414	-1.91858
H	-2.23023	-2.15817	-2.1935

TS$_{diol}$

O	-0.33149	1.28669	1.10868
C	-1.26731	0.35469	-1.03301
C	-1.70084	-0.72237	-0.36116
O	-0.88744	-1.11341	0.67307
C	0.13635	-0.13966	0.75726
H	1.04624	-0.51533	1.20489
C	-0.04668	0.82524	-0.34207
H	0.7564	1.38264	-0.80567
O	1.94128	0.30035	0.15978
H	2.81632	0.49272	0.5119
H	1.02233	1.08307	0.72577
C	-5.76447	-1.1874	-2.98181
C	-3.81566	-0.92676	-1.55947
C	-3.37179	0.15123	-2.36825
C	-4.15178	0.54387	-3.49018
C	-5.32108	-0.11161	-3.78878
C	-3.03446	-1.31904	-0.43733
C	-2.15691	0.81068	-2.03432
H	-3.79878	1.38076	-4.11104
H	-5.92694	0.18954	-4.65603
H	-1.81991	1.64566	-2.6668
O	-3.49327	-2.4067	0.36975
C	-4.22503	-1.89633	1.48732
H	-5.16341	-2.40509	1.56161
H	-3.66232	-2.05319	2.38378
H	-4.3969	-0.84883	1.35278
O	-5.03043	-1.58666	-1.89261
O	-6.98642	-1.84897	-3.31956

P$_{diol}$

C	2.63025	-3.02509	1.11868
C	3.87255	-3.11436	0.50393
C	4.36591	-4.39331	0.20092
C	3.61997	-5.55226	0.51263
C	2.35644	-5.48584	1.13671
C	1.90224	-4.20219	1.42071
H	6.23461	-3.72355	-0.68576
H	4.45339	-2.23398	0.26013
C	5.64501	-4.60001	-0.43748
C	5.35539	-7.04235	-0.41072
C	6.10785	-5.83492	-0.72339
H	7.06399	-5.99497	-1.20087
O	4.09267	-6.81405	0.22062
O	5.68053	-8.19765	-0.6252
O	1.72758	-6.67527	1.38947
C	0.40761	-6.66838	2.04003

H	-0.32894	-6.13447	1.43501
H	0.14642	-7.72301	2.11853
H	0.45716	-6.21041	3.03072
O	0.68839	-3.86212	2.03346
C	0.67896	-2.448	2.10502
C	1.80491	-1.91724	1.57661
H	2.32853	-1.35687	2.32273
H	0.60013	-2.15057	3.12983
O	1.47265	-1.06931	0.47411
H	1.3697	-0.1665	0.78384
O	-0.45315	-1.95166	1.38608
H	-1.07195	-1.55002	2.00039

TS_{r1}

C	1.38956	-0.84857	0.30489
C	0.39152	-1.77577	0.12071
C	-0.94177	-1.32654	0.04002
C	-1.22124	0.05139	0.1579
C	-0.2045	1.01221	0.32507
C	1.09197	0.51268	0.38958
H	-1.8737	-3.27029	-0.22373
H	0.6171	-2.83265	0.03587
C	-2.06407	-2.20519	-0.1359
C	-3.61838	-0.31539	-0.0806
C	-3.32929	-1.73469	-0.19206
H	-4.18749	-2.37883	-0.32562
O	-2.49965	0.51557	0.10892
O	-4.69391	0.21454	-0.13459
O	-0.42324	2.34394	0.48559
C	-1.11298	3.03714	-0.56884
H	-2.16545	2.75604	-0.60566
H	-1.01871	4.09544	-0.33027
H	-0.63679	2.83828	-1.53438
O	2.20481	1.29333	0.54515
C	3.42767	0.70059	0.22974
C	2.81326	-1.24072	0.50622
H	3.04396	-1.55078	1.52981
H	4.21555	1.03528	0.9065
O	3.45002	-2.169	-0.33447
H	3.48206	-1.7722	-1.21447
O	3.80191	0.82023	-1.11255
H	4.07331	1.72807	-1.2828

P_{r1}

C	1.38956	-0.84857	0.30489
C	0.39152	-1.77577	0.12071
C	-0.94177	-1.32654	0.04002
C	-1.22124	0.05139	0.1579

C	-0.2045	1.01221	0.32507
C	1.09197	0.51268	0.38958
H	-1.8737	-3.27029	-0.22373
H	0.6171	-2.83265	0.03587
C	-2.06407	-2.20519	-0.1359
C	-3.61838	-0.31539	-0.0806
C	-3.32929	-1.73469	-0.19206
H	-4.18749	-2.37883	-0.32562
O	-2.49965	0.51557	0.10892
O	-4.69391	0.21454	-0.13459
O	-0.42324	2.34394	0.48559
C	-1.11298	3.03714	-0.56884
H	-2.16545	2.75604	-0.60566
H	-1.01871	4.09544	-0.33027
H	-0.63679	2.83828	-1.53438
O	2.20481	1.29333	0.54515
C	2.20466	2.26039	-0.5265
C	2.88764	-1.00571	0.47275
H	3.21413	2.46957	-0.81306
O	3.5244	-1.93399	-0.36794
O	1.5861	3.44639	-0.11796
H	2.09713	3.83585	0.59899
H	1.67608	1.85758	-1.3651
H	3.43376	-0.43731	1.19634

TS_{r2}

O	-4.04302	-0.59074	0.97385
C	-1.45394	-1.1608	-0.14972
C	-1.3783	0.27743	-0.26717
O	-2.51892	0.8794	-0.38593
C	-3.70432	-0.36879	-0.23279
H	-4.3981	-0.01524	-1.0006
C	-2.78452	-1.52703	-0.30074
C	3.43324	0.06606	0.05136
C	1.00573	0.10659	-0.10377
C	0.93634	-1.32427	0.00592
C	2.18097	-2.03407	0.13405
C	3.36161	-1.38466	0.15427
C	-0.121	0.91899	-0.24679
C	-0.29824	-1.94657	0.00122
H	2.14701	-3.1156	0.21722
H	4.31054	-1.89373	0.25121
H	-0.36949	-3.02398	0.09485
O	2.19975	0.73699	-0.07599
O	0.04225	2.25337	-0.41564
C	-0.6583	3.11286	0.50198
H	-0.35948	2.89443	1.53178

H	-0.35256	4.12458	0.24214
H	-1.73814	3.00552	0.39507
O	4.42261	0.73885	0.06642
H	-2.81356	0.34426	1.0092
H	-2.90479	-2.00847	-1.24871
O	-3.12917	-2.45155	0.73434
H	-3.64495	-3.17093	0.36278

P_{r2}

C	1.38956	-0.84857	0.30489
C	0.39152	-1.77577	0.12071
C	-0.94177	-1.32654	0.04002
C	-1.22124	0.05139	0.1579
C	-0.2045	1.01221	0.32507
C	1.09197	0.51268	0.38958
H	-1.8737	-3.27029	-0.22373
H	0.6171	-2.83265	0.03587
C	-2.06407	-2.20519	-0.1359
C	-3.61838	-0.31539	-0.0806
C	-3.32929	-1.73469	-0.19206
H	-4.18749	-2.37883	-0.32562
O	-2.49965	0.51557	0.10892
O	-4.69391	0.21454	-0.13459
O	-0.42324	2.34394	0.48559
C	-1.11298	3.03714	-0.56884
H	-2.16545	2.75604	-0.60566
H	-1.01871	4.09544	-0.33027
H	-0.63679	2.83828	-1.53438
O	2.20481	1.29333	0.54515
C	2.88764	-1.00571	0.47275
O	3.5244	-1.93399	-0.36794
H	3.55644	-1.53719	-1.24794
H	2.1403	1.78198	1.36896
C	3.21236	-1.44213	1.91348
O	4.3617	-1.64762	2.29174
H	2.33745	-1.5585	2.58736
H	3.3379	-0.04931	0.30702
